# Attachment-Related Differences in Emotion Regulation in Adults: A Systematic Review on Attachment Representations

**DOI:** 10.3390/brainsci13060884

**Published:** 2023-05-31

**Authors:** Dirk W. Eilert, Anna Buchheim

**Affiliations:** Institute of Psychology, University of Innsbruck, 6020 Innsbruck, Austria; anna.buchheim@uibk.ac.at

**Keywords:** attachment representation, adult attachment interview, adult projective picture system, emotion regulation, SCL, HRV, neuroimaging, oxytocin, cortisol, facial expressions

## Abstract

In recent years, there has been an increase in the prevalence of mental disorders connected with affective dysregulation and insecure attachment. Therefore, it is even more important to understand the interplay between an individual’s attachment representation and patterns of emotion regulation. To our knowledge, this is the first systematic review to examine this association. PsycInfo, PsyArticles, and PubMed were searched for studies that examined attachment-related differences in emotion regulation in adults. To examine the unconscious attachment representation, only studies using the Adult Attachment Interview or the Adult Attachment Projective Picture System were included. Thirty-seven peer-reviewed studies (with a total of 2006 subjects) matched the PICO criteria. Emotion regulation was measured via four objective approaches: autonomic nervous system, brain activity, biochemistry, or nonverbal behavior. Across all measurements, results reveal a significant correlation between attachment representation and emotion regulation. Secure attachment correlates consistently with balanced emotion regulation, whereas it is impaired in insecure and dysfunctional in unresolved attachment. Specifically, unresolved individuals display counterintuitive responses and fail to use attachment as a resource. Insecure-dismissing attachment is associated with an emotionally deactivating strategy, while on a physiological, biochemical, and nonverbal level, emotional stress is still present. There is still a lack of studies examining preoccupied individuals. In addition to interpreting the results, we also discuss the risk of bias, implications for psychotherapy and coaching, and an outlook for future research.

## 1. Introduction

A meta-analysis [1] involving a total of 308,849 subjects showed that the absence of genuine and deep attachments to others—the feeling of loneliness—increases the likelihood of death more than other risk factors, such as air pollution, high blood pressure, obesity, lack of exercise, excessive alcohol consumption, or even smoking. Or, put the other way around, if we have strong interpersonal relationships, the probability that we will survive increases by 50 percent compared to when we feel lonely. These are impressive and, at the same time, alarming findings that underscore the enormous importance of interpersonal relations. The strong impact of social connectedness, especially on our health and general wellbeing, has also been confirmed with further meta-analyses [2].

However, what does “feeling lonely” actually mean? The mere presence of other people does not make us feel connected and secure. Sometimes, we are surrounded by people (e.g., on the train) but still feel lonely. Likewise, we can be alone in a mountain hut but still feel connected to others—to people who are physically not present in the latter case. This is where a person’s individual attachment representation plays a role. A variety of studies showed that secure attachment is associated with a lower sense of loneliness, and insecure attachment patterns are positively correlated with a sense of loneliness [3,4,5]. A secure attachment representation provides the inner foundation for our ability to form fulfilling relationships with others and to feel a sense of social connection, even and especially in moments of aloneness. Attachment insecurity, on the other hand, undermines this ability and turns moments of aloneness into the undesirable experience of loneliness (for a review, see [6]). This link may be because a person’s attachment representation serves as a perceptual filter for current relationship experiences. Thus, an individual’s attachment representation—the inner working model of attachment—accounts for biasing how the person perceives interpersonal interactions. We tend to view current relationships in light of the past and preferentially perceive the information that confirms our existing interpersonal beliefs and expectations [7,8]. In this way, a sense of secure attachment supports the feeling, even if the person is alone right now, that the partner and/or friends will be by the person’s side if he/she needs them emotionally. This ensures that a moment of being alone can be enjoyed, full of inner security that others are benevolently available. Furthermore, Mikulincer and Shaver [9] summarized a large number of studies that have shown that attachment insecurity is associated with detrimental effects on the perceived stability and quality of interpersonal relationships in both adolescence and adulthood. Another possible reason that a sense of secure attachment fosters a feeling of social connection, even when the person is alone, is that secure attachment is related to positive self-esteem [10]. Indeed, the research results indicate that self-esteem and the feeling of loneliness influence one another in a reciprocal manner, e.g., [11]. The conclusion from all these research findings is that there are many studies that show the interaction between attachment representation on the one hand and personal feelings of loneliness or social connectedness, respectively, on the other. This shows the connection of a person’s attachment representation to health and general wellbeing. This link was also confirmed with a meta-analysis [12], which concluded that a secure attachment is the core element of positive adaptation in terms of resilience.

Recognizing what an important foundation secure attachment is for a resilient and flourishing life raises the question of what is at the origin and core of the developing self, and thus, at the core of mental attachment representation. The Strange Situation Procedure [13] makes it possible to already assess the quality of the attachment between child and mother (another important attachment figure) at the age of 11 to 15 months—a developmental age at which children usually already speak their first words but which is, nevertheless, primarily preverbal. It was John Bowlby, the “father of attachment theory”, himself, who emphasized the importance of social preverbal experience in the development of the internal working model of attachment ([14], p. 157): “During the earliest years of our lives, indeed, emotional expression and its reception are the only means of communication we have, so that the foundations of our working models of self and attachment figure are perforce laid using information from that source alone”. Both Bowlby and Ainsworth have stressed that attachment implies strong affect and regulation, and by this, they meant the complete spectrum of human emotions from anger through sadness and fear up to love and compassion [13]. From these considerations, it can be concluded that the origin as well as the core of the developing self and attachment representation are emotional in nature. Thus, mental attachment representations are shaped by emotions and the process of emotion regulation. This explains the association between attachment and emotion regulation processes.

The goal of this systematic review is to further understand attachment representation at its emotional core by summarizing the current state of research on the topic of attachment-related differences in emotion regulation. This is conducted with two superordinate goals in mind. First, to understand even better the individual factors for healthy and balanced emotion regulation. This is an important point, especially in the current times. In fact, one study showed that in 2020, due to the COVID-19 pandemic, the prevalence of anxiety disorders and depression increased by more than 25 percent worldwide [15]. This shows the need to broaden and deepen our understanding of emotion regulation even more. Secondly, a meta-analysis showed that problematic levels of loneliness are experienced by a substantial proportion of the population in many countries [16]. Therefore, we see it as meaningful to contribute to the understanding of how specific psychological conditions contribute to people feeling lonely despite increasing digital connectivity. For this, a person’s attachment representation takes on a key function, as explained above. Since the feeling of loneliness is an emotional phenomenon, the link between individual attachment representation and emotion regulation is particularly promising here.

### 1.1. Two Perspectives on the Measurement of Attachment

According to Bowlby [17,18,19], infants internalize interactions with their primary attachment figures and, in this way, form an inner working model of attachment that includes mental representations of both self and attachment figures. This inner working model of attachment is not fixed but malleable and can be changed, for example, through new resourceful relational experiences (also in the context of psychotherapy) over the course of life and, likewise, through targeted interventions (e.g., [20,21,22]).

Measurement of individual differences in attachment patterns began in the 1960s with Mary Ainsworth’s analysis of attachment qualities between mothers and children in Uganda [23]. She concluded that children’s attachment quality can be classified into three categories of organized attachment: secure, insecure-avoidant, and insecure-ambivalent [13]. Main and Solomon [24] later discovered a fourth classification to these three: disorganized. From there, two different perspectives developed in attachment research; one could say two different cultures emerged (for a large review, see [25]). Both developmental strands are based on the Ainsworth coding system of the Strange Situation Procedure.

On the one hand, the social psychological approach, beginning with Hazan and Shaver [26] in the late 1980s, measured attachment in romantic relationships via self-report questionnaires. Based on these first self-report questionnaires, a variety of other measurement instruments developed that remained mainly in line with the self-assessment approach in the social and personality psychology tradition (for a review, see [27]). On the other hand, in 1985, George et al. [28] and Main et al. [29] developed the Adult Attachment Interview (AAI), which they called “a move to the level of representation” (as distinct from the assessment of child behavior in the Strange Situation Procedure). This was the beginning of the history of research in the measurement of attachment in adults.

The AAI [28] is a well-validated semi-structured interview of 20 questions asked in a set order with standard probes intended to elicit thoughts, feelings, and memories about early attachment experiences, including experiences of separation, rejection, loss, and abuse. The interview also asks subjects to reflect on their parents’ styles of parenting and how this influenced their development and life as adults. Its scoring system (e.g., coherence, idealization, derogation, and anger) is designed to quantify the individual’s current state of mind with respect to childhood attachment relationships. Main and Goldwyn [30] identified three major organized patterns of adult attachment that can be derived from these responses: (1) secure/autonomous, (2) dismissing, (3) preoccupied, (4) unresolved, and cannot classify. Compared to the organized categories (secure, dismissing, and preoccupied), which involve the capacity to mobilize a consistent strategy for approaching attachment-related memories, those with unresolved attachment are characterized by failure to resolve attachment-related trauma (e.g., loss or abuse) and dissociative defenses for coping with attachment stress. Psychometric properties are excellent (see [31]).

The Adult Attachment Projective Picture System (AAP) developed by George and West [32] is also in this narrative (observation) assessment tradition of developmental psychology. The AAP is a well-validated measure that assesses adult attachment mental representation. The AAP is based on the analysis of “story” responses to a set of theoretically derived attachment-related drawings of scenes depicting solitude, illness, separation, death, and potential maltreatment. Drawings portray adults and children alone (three monadic pictures representing abandonment) as well as adult–adult/adult–child dyads (four dyadic pictures representing interpersonal distress) and one neutral picture. Individuals are asked to tell a story about each picture following a standardized set of interview probes. The classification is derived by evaluating the response patterns for the whole set of seven picture stimuli, each response of which is evaluated for content, discourse, and defensive processes. Organized attachment is defined in the AAP, following the attachment literature at large, as secure, insecure-dismissing, and insecure-preoccupied classifications. Transcripts are judged unresolved when there is no evidence of representational resolution of frightening elements in at least one story. The AAP has demonstrated solid psychometric properties, including test–retest reliability, inter-judge reliability, discriminant validity [32,33,34,35,36], and convergent validity with the AAI [32,34,37].

In sum, both the AAI and AAP use four classifications of attachment representation in adults, analogous to Ainsworth’s four categories for attachment quality in children: secure, insecure-dismissing, insecure-preoccupied, and unresolved. The first three correspond to organized attachment representations, and the last to the disorganized one.

Meta-analyses (e.g., [38]) could show that there is only a trivial to small overlap between these two approaches—self-report questionnaires versus assessment by narrative interview—to measure attachment. One possible rationale is that those self-report questionnaires measure conscious appraisal processes, whereas assessing attachment representation via an interview (e.g., AAI or AAP) measures unconscious processes (see [39]). Furthermore, self-report questionnaires cannot detect when psychological defenses bias responses. In addition, Mikulincer and Shaver ([27], p. 92) write that they would acknowledge that there are inner working models of attachment, which are measurable, but they emphasize that these cannot be directly assessed by the major self-report attachment scales. This was already emphasized by Bowlby [17], according to whom the attachment system can only be observed under conditions in which it is emotionally activated. The interview procedure of the AAP (due to the AAP picture stimuli), for example, achieving exactly this activation during the task, has already been shown by Buchheim et al. [40] in a neuroimaging study.

Since this review is designed to analyze the relationships between unconscious attachment representation and emotion regulation, we will focus the following analysis on the two most widely used representational attachment measures, the AAI, and the AAP. Another reason for this emphasis is that despite extensive searching in research databases, we were unable to locate a systematic review that examined the relationship between narratively assessed attachment representations (measured via AAI or AAP) and patterns in emotion regulation. Therefore, this systematic review is intended to fill this research gap. For a review of the correlation between self-reported attachment style and emotion regulation, see Mikulincer and Shaver [41].

Building on the above notions of Bowlby and Ainsworth’s attachment theory and definitions of attachment classifications [30,32], the following hypotheses are formulated: Individuals with insecure-dismissing attachment representations tend to downregulate their emotional responses to deactivate, as much as possible, their need for attachment at the emotional level. In contrast, individuals with insecure-preoccupied attachment representations tend to upregulate their emotional responses to communicate their need for attachment more clearly to the outside world. Securely attached individuals, on the other hand, will exhibit balanced emotion regulation. They may well show stress in response to attachment-relevant stressors, but it becomes clear that they cope emotionally with the stress while remaining in touch with their emotional resources. Because attachment disorganization reflects the lack of any organized attachment strategy as well as any emotion regulation strategy, the response of individuals with an unresolved attachment representation is quite different. Here, emotion regulation will tend to be dysfunctional, that is, the stress response will be the strongest compared to the three organized attachment representations. We hypothesize that these patterns of emotion regulation will be evident in all the types of emotional response described below—in the four approaches to an objective measurement of emotion.

### 1.2. Four Approaches to the Objective Measurement of Emotion (Regulation)

Before describing what is meant by emotion regulation and how it can be measured, it is first necessary to define the term emotion. According to Matsumoto and Hwang [42], emotion is defined as a “transient, bio-psycho-social reaction to events that have consequences for our welfare and potentially require immediate action”. Emotions are biological because they lead to specific changes in the brain, for example, in the limbic system and in the autonomic nervous system. Emotions are psychological because they involve cognitive processes, such as an activated approach or avoidance motivation, or an opening or narrowing of perception. The social component involves two aspects: On the one hand, emotions play a central role in social interaction, and on the other hand, we express them through facial expressions and body language to the outside world to (unconsciously) communicate them to our social environment.

This definition includes three key features [43]: First, emotions are triggered only in response to a situation that the person evaluates as relevant to his or her own goals. Second, emotions nudge change at different interrelated levels. They drive physiological, cognitive, and behavioral modifications. The third key feature relates to the heart of emotion regulation, namely the notion that emotions are malleable. Thus, although they can strongly influence our decisions and actions [44], they can still be effectively influenced by sometimes simple techniques. For example, Firk et al. [45] showed that in mothers who were played the recorded crying of their babies as a stress stimulus while they were lying in an fMRI scanner, the amygdala was activated. In contrast, if the mothers now performed a mental task at the same time (they counted the dots on a picture), this immediately downregulated the amygdala. The subjects also felt less emotionally stressed.

**Figure 1:**
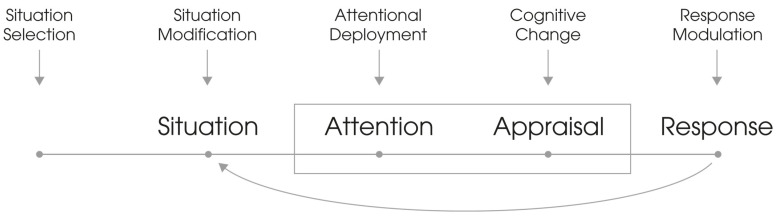
Adapted Process Model of Emotion Regulation, see Gross [43].

Because these three key features are found in many theories of emotion, Gross [43] refers to them as the consensual model of emotion, which is also called the process model of emotion regulation (see also [46]). Figure 1 shows, following Gross [43], in schematic form, the sequence of situation, attention, appraisal, and response (with the organismal “black box” between situation and response) given by the process model of emotion regulation. The figure also shows, assigned to the individual elements of the sequence, five possible approaches to emotion regulation.

According to this model, each emotion is triggered by a situation that is psychologically relevant to the person. This also means that the focus of attention—consciously or just unconsciously initiated—shifts to the emotional trigger. Now, this follows the cognitive–emotional appraisal of the situation, which then leads to a response, whereby the response evoked by the emotion, as already described, manifests itself on physiological, cognitive, and behavioral levels. As a result, this response often changes the situation itself. If, for example, we make a mistake or break a rule and then blush, others do not perceive the mistake so badly and judge us more positively (e.g., more likable and more trustworthy). Blushing, thus, has an appeasing effect and, in certain situations, even ensures that others see us in a more positive light [47].

In accordance with Gross [43], emotion regulation “refers to the processes by which we influence which emotions we have, when we have them, and how we experience and express these emotions”. To include all the approaches of emotion regulation shown in Figure 1 in this systematic review, we do not focus on an examination of the specific strategies of emotion regulation but on the analysis of differences in emotional responses in the face of attachment-related stressors that are linked to variation in individual attachment representations. Following Mauss and Robinson [48], with regard to the modal model of emotion, five ways can be considered to measure emotional responses: subjective experience (via self-report), peripheral physiology (ANS), affect-modulated startle, central physiology (CNS), and (nonverbal) behavior. A sixth measurement possibility, emphasized by Baum et al. [49], is the inclusion of the biochemical level.

In this review, the analysis is limited to objective measures of emotional response, thus excluding the measurement of subjective response using self-report questionnaires. One reason to exclude self-report questionnaires for assessing emotional experience is that striving for social desirability can bias the answers, but the more critical reason at this point is that, according to studies, about one in ten people in the population has difficulty perceiving and describing their own feelings in words [50]. This is called alexithymia, which means “no words for feelings” [51]. Studies suggest that individuals who meet the criteria for alexithymia are less able to accurately report their feelings in self-report questionnaires [52]. Among other characteristics, divorce and singleness were associated with higher alexithymia scores. Since the results of a study indicated that anxious and avoidant attachment styles significantly predicted a history of both divorce and singleness versus a partnered relationship status [53], it is important to consider this context here.

Because for measuring the startle reflex, the most robust component of the behavioral cascade is the eye blink, and this can be counted as a facial expression, we include this measurement type in the behavioral category. This leaves a total of four approaches to the objective measurement of emotional response that we consider for this systematic review. These are each briefly outlined below. We thereby concentrate on those pieces of information that enable the reader to understand the subsequent conclusions regarding the findings of this systematic review.

A preliminary note: Each of the following measurement approaches of emotions is sensitive to other emotional dimensions. The most commonly assumed dimensions are valence, arousal, and approach-avoidance motivation. Valence refers to the qualitative way emotion is felt in terms of unpleasant versus pleasant. Arousal reflects the psychophysiological activation level, i.e., relaxed versus aroused. Approach-avoidance motivation finally looks at the direction of motivation in terms of moving towards something or moving away from something. In addition to the dimensional view, there is a second way of looking at emotions: the discrete emotion perspective. This point of view assumes that each emotion (e.g., fear, anger, or joy) has a unique underlying profile in subjective experience, physiology, and behavior, see, (e.g., [54]).

#### 1.2.1. First Approach: Peripheral Physiology (ANS)

The autonomic nervous system (ANS) can be divided into three branches: the sympathetic, parasympathetic, and enteric nervous systems ([55], p. 158). For the following analysis, the first two are of importance. While the sympathetic nervous system becomes active when our arousal increases, the parasympathetic nervous system dominates during periods of rest. Basically, the ANS response reveals the arousal level of a person. A meta-analysis of Cacioppo et al. [56] indicates that some ANS responses (e.g., blood pressure) may provide complementary clues to emotional valence. However, a meta-analysis by Behnke et al. [57] showed that positive emotions produce no or only weak and highly variable increases in ANS reactivity. This suggests that only negative valence can be detected using ANS measurements. Furthermore, it was impossible to differentiate the positive emotions regarding their ANS responses.

Research [56] showed that certain physiological parameters tended to reflect sympathetic or parasympathetic activation, respectively, or, for some indicators, the activity of both. For example, skin conductance level (SCL) indicates primarily sympathetic activation, whereas measures of heart rate variability (HRV)—such as RMSSD (root mean square of successive differences), peak-valley, or HF-HRV (high frequencies HRV)—are correlated to parasympathetic activity (also called vagal tone) (for an overview, see also [58,59]). In contrast, heart rate (HR) and blood pressure (BP) reflect the combined activity of both branches of the autonomic nervous system.

In a meta-analysis [60], researchers tried to find out how different emotions are differentiated by their responses in the autonomic nervous system. However, individual emotions could not be clearly differentiated based on ANS responses, such as heart rate, blood pressure, or skin conductance. It was not possible to identify specific physiological “fingerprints” for certain emotions. Thus, according to current research, ANS measurements reveal a person’s arousal level (and possibly also the negative valence of an emotional state), but, currently, specific emotions cannot be clearly distinguished.

#### 1.2.2. Second Approach: Central Physiology (CNS)

Panksepp [54], among others, have theorized that emotions are reflected not only in peripheral physiology (ANS) but also in central nervous system (CNS) activity. To investigate the association between CNS activity and emotional states, researchers use EEG and neuroimaging methods. These two approaches generate different types of data. Therefore, they will be considered subsequently, one after the other.

For the analysis of the correlations between emotional states and brain activity, frontal asymmetry is of particular interest with respect to EEG measurements. Emotional processes—so research assumed for a long time—should predominantly take place in the right hemisphere (e.g., [61]). However, recent research has differentiated this even further. Thus, for emotions characterized by an avoidance motivation (e.g., fear, shame), the prefrontal cortex (PFC) of the right hemisphere plays a major role, whereas, for emotional states with a predominant approach motivation (e.g., joy, anger), the left PFC is more active [62,63,64]. Thus, according to current research, EEG measurements related to frontal asymmetry primarily reflect the momentary direction of motivation, namely approach (left hemisphere) versus avoidance (right hemisphere) motivation.

Neuroimaging studies have the advantage over EEG measurements in that they can localize brain activity more concretely. Frequently used methods are fMRI (functional magnetic resonance imaging), which measures the uptake of oxygen in the blood, and PET (positron emission tomography), which assesses the metabolic activity in the brain via injection of a radioactive isotope. Even though research is making progress in identifying specific neuronal networks for certain emotions, clear results are still lacking. In their review, Mauss and Robinson [48] conclude that neuroimaging measurements are currently not suitable for identifying specific emotions according to the discrete emotion perspective. However, current research supports the idea that neuroimaging studies provide clues to the motivational direction activated. For example, Wager et al. [65] showed in their meta-analysis that there is a systematic relationship between avoidance motivation and activity in the amygdala and anterior cingulate cortex (ACC). Furthermore, they found a correlation between approach-motivated states and activity in the anterior medial PFC. Thus, it can be concluded that neuroimaging measures can primarily provide evidence for approach versus avoidance motivation.

Another aspect that neuroimaging studies can further reveal is the efficiency of neural emotion regulation networks in response to stressors. For example, one such neural circuit of emotion regulation is the frontoparietal network, which includes the dorsolateral prefrontal cortex (dlPFC) and the posterior part of the parietal lobe. Some researchers even refer to this network as the “immune system of the mind” [66]. In one study [67], researchers were able to directly increase subjects’ emotional resilience by activating the dlPFC via transcranial direct current stimulation. Furthermore, Vrtička and Vuilleumier [68] and Lenzi et al. [69] demonstrated that the activity of the frontolimbic system intervenes in modulating social and emotional behaviors and affect-regulating functions that are specifically involved in the attachment system.

#### 1.2.3. Third Approach: Biochemical Level

Baum et al. [49] suggest incorporating biochemical measurements (e.g., hormones or neurotransmitters) into the study of emotional responses. In current studies, oxytocin and cortisol in particular play a key role in investigating the relationship between attachment representation and emotion regulation.

The hypothalamic–pituitary–adrenal (HPA) axis is one of the major physiological stress response systems whose end product is cortisol [70]. Nevertheless, not every form of stress causes cortisol to be released. A meta-analysis [71] showed that experimental tasks containing both uncontrollable and social evaluative elements were associated with the largest cortisol changes. The social component indicates the importance of cortisol in the response to attachment-related stressors. In addition, the results of one study indicate a relative increase in right frontal activity with cortisol [72]. This suggests that cortisol indicates avoidance motivation. This hypothesis is supported by further research [73,74], which has demonstrated that elevated cortisol levels make us more conservative in our decision making (for example, in financial investments) and equally less willing to take risks. Similarly, many emotions associated with avoidance motivation are associated with increased cortisol release, e.g., anxiety [75] and shame [76].

Oxytocin plays a central role in the experience and behavior in interpersonal relationships, especially in prosocial action. The prosocial influence of oxytocin points to its evolutionary role in mammalian social bonding and attachment [77]. Thus, many studies have already demonstrated that oxytocin improves empathy (e.g., [78,79]). In addition, oxytocin specifically also promotes communication in a partnership; If a conflict occurs in a relationship, an increased oxytocin level ensures that the partners communicate with each other in a more positive and appreciative manner—for example, they show less contempt but more signals of emotional closeness such as eye contact [80]. At the same time, the level of the stress hormone cortisol was lower after the dispute, which, in addition to the more positive communication, can also be attributed to the fundamental anxiety- and stress-relieving effect of oxytocin on the amygdala [81]. In addition, oxytocin is associated with many prosocial emotions characterized by approach motivation, such as compassion [82] and both romantic and maternal love [83]. All these research findings indicate that oxytocin correlates with its prosocial function with approach motivation.

Overall, based on the current state of research, it can thus be concluded that biochemical measurements can indicate approach or avoidance motivation.

#### 1.2.4. Fourth Approach: Nonverbal Behavior

The analysis of emotions in nonverbal behavior originated in the 19th century in the work of Charles Darwin [84]. Darwin already emphasized that emotions are multimodal phenomena. They are manifested not only in one nonverbal channel, such as facial expression, but in several different ones, such as gestures, posture, gait, voice, and touch. Recent studies also confirm this view [85]. By taking a multimodal view of emotions, cross-cultural expressions of different emotions have been discovered in recent years. For example, pride and love do not differ from joy in pure facial expressions. Only when at least the tilt angle of the head is also considered do the two emotions show up in differentiation from joy as a nonverbal expression [86,87]. According to Eilert [88], nonverbal behavior can be divided into a total of eight observation channels: facial expressions, head posture, gestures, foot–leg behavior, body posture, psychophysiology, voice, and interpersonal movement behavior. In analyzing the emotional response, facial expressions play a key role in the “stage of emotions”. This is supplemented by signals from the voice.

Studies showed that the emotions revealed in facial expressions coincide with the subjective experience of this emotion, physiological responses in the body, and, subsequently, observed behaviors (for an overview, see [89]). Thus, facial expressions are the only response system that can not only provide emotional dimensional cues but also allow us to infer specific emotions such as fear, joy, or anger. In 1978, the Facial Action Coding System (FACS) was published by Ekman and Friesen [90] and is the most widely used scientific coding system for measuring and describing facial expressions today (for an overview, see [91]). Based on anatomy, the FACS describes 44 so-called action units (AU) and their exact facial recognition characteristics. Action units are specific facial movements, usually caused by one muscle, but sometimes by the combination of several muscles. For example, eyebrow contraction is action unit 4. Finally, certain combinations of action units in their interaction produce a prototypical facial expression by which certain emotions can be inferred cross-culturally. For example, the action unit sequence AU1 + 2 + 4 + 5 + 7 + 20 shows the facial expression of fear (for an overview of different emotions, see [85]).

Another behavior coding system that incorporates the FACS is the Specific Affect Coding System (SPAFF). It was developed by Gottman and Krokoff in 1989 for the purpose of systematically observing affective behavior in the context of marital conflict [92]. Unlike the FACS, it directly assigns specific codes to particular affective states (e.g., affection, contempt, defensiveness, or stonewalling). Because the SPAFF is designed to code behavior in situations of interpersonal communication, it may be particularly informative for assessing emotional responses in terms of differences in attachment representations.

As stated above, we count the startle reflex, measured by eye blink, as facial behavior. According to research, the startle response is a marker of negative emotional valence, especially reliable in the context of high-arousal negative stimuli (e.g., [93]). Often the startle reflex is measured by facial electromyography (EMG). EMG is another way to measure facial behavior objectively and reliably. Here, the electrical potential of the facial muscles is measured by electrical sensors placed on the face. The two most common muscles that are examined are the corrugator supercilii (which enables us to contract the eyebrows) and the zygomatic major (which pulls the corners of the mouth up into a smile). Research by Larsen et al. [94] indicates that activity of the corrugator supercilii muscle is related to negative emotional valence, while contraction of the zygomatic major muscle is associated with positive emotional valence.

Finally, the voice is considered. The agreement rates for assigning a specific vocal pattern to a specific emotion are, on average, only 54 to 70 percent within a culture—and typically only between 32 and 64 percent across cultures [95]. The voice can thus only reveal something about concrete emotions in individual cases. It should rather be considered as a marker for arousal. In the channel of the voice, besides the content of the words, two sub-channels can be distinguished: the tone of voice and the style of speech. The two most important properties of vocal tone are pitch (also referred to as fundamental frequency or F_0_) and loudness. The change in pitch basically indicates the arousal of the person [96] as follows: if the pitch becomes lower, this signals a decrease in arousal; if the pitch increases, this indicates increased arousal. A louder voice also typically indicates increased arousal, while a quieter voice indicates low arousal levels [97]. Regarding the style of speech, here, we only consider the speech rate. The speech rate also basically indicates the arousal of the person in which a slow rate rather signals a low arousal level, and a faster one a higher arousal (e.g., see [98,99]).

Finally, following Mauss and Robinson [48] as well as Baum et al. [49], Table 1 shows an overview of all findings in this section.

### 1.3. Different Types of Emotional Stress

The types of stress that people face in their lives can be broadly divided into two categories [41]: first, attachment-related stressors, such as separation or loss through death; second, attachment-irrelevant stressors, i.e., threats that do not activate a person’s attachment system, such as fear of exams or psychological stress caused by accidents or natural disasters. In the following, this systematic review focuses on analyzing patterns of emotion regulation in relation to attachment-relevant stressful events. Thus, not only during the assessment of individual attachment representation is the emphasis on activation of the attachment system but also in the triggering of emotion regulation. Because according to Mikulincer and Shaver [41], differences in individual attachment representations are particularly critical to understanding individual differences in the way people experience and react to attachment-related triggers for stress.

## 2. Methods

This systematic review was prepared and conducted according to the Preferred Reporting Items for Systematic Reviews and Meta-Analyses (PRISMA) recommendations [100]. Screening and data extraction were completed using DistillerSR (Version 2.43). Both screening phases, as well as data extraction and quality assessment of the included studies, were performed by both authors to reduce the error rate and possible bias. Disagreements were resolved by a discussion between the authors. A qualitative synthesis was chosen to summarize the results due to the heterogeneity of the studies.

### 2.1. Search Strategy

To conduct the entire search, a search protocol was written beforehand, based on which the search was then conducted. Four search strategies were used to identify potential studies for inclusion in this systematic review.

First, a systematic literature search was conducted in PsycInfo, PsyArticles, and PubMed on 14 March 2023. We used the same search strategy for all three databases. The design of the search syntax was guided by the PICO approach. The search syntax included the following search key terms (see Table 2). The different PICO components were connected with the Boolean operator AND. The search settings were chosen so that any field was searched (e.g., title, abstract, and keywords).

Second, the second author’s publication list was searched on the Internet (https://www.uibk.ac.at/de/psychologie/mitarbeiter/buchheim/publications/, accessed on 15 March 2023) for other conducted studies eligible for inclusion in this systematic review. Subsequently, based on her years of experience in attachment research, the second author conducted a hand search of PubMed on 16 March 2023 to identify additional studies potentially to be included according to the pre-specified inclusion and exclusion criteria. Fourth, backward citation chaining in two review articles [41,101] was used to determine whether additional studies should be included in this systematic review.

### 2.2. Inclusion and Exclusion Criteria (Eligibility)

To determine the inclusion and exclusion criteria in the pre-specified search protocol and, thus, assess the eligibility of studies for inclusion in this systematic review, the PICO approach was used (see Table 3).

The following were defined as exclusion criteria: (a) studies with children (<12 years), (b) in which the attachment style was measured with self-report questionnaires (see introduction section for rationale), or (c) in which the outcome of emotion regulation was measured via a self-assessment of the subjects. We also decided to apply the last exclusion criterion, in addition to the reasons already mentioned above, because research (e.g., [102,103]) has shown that insecure-dismissing individuals tend to be psychobiologically incoherent. That is, they show a discrepancy between self-assessed feelings and objective measures of emotional response (e.g., based on facial expressions or ANS responses).

### 2.3. Quality Assessment of Included Studies

To assess the risk of bias in the included studies, a modified checklist of four items derived from Busse and Guyatt [104] was applied. As mentioned above, the quality assessment of the studies was performed independently by both authors. This was to reduce the risk of potential bias in the assessment of the risk of bias.

The quality assessment checklist included the following items: (a) representativeness of the sample for the population of interest; (b) adequacy of dropout rate (<25%); (c) adequacy of the missing data rate (<10% within measurements); and (d) reliability and validity of the measurement methods used. Each item was rated with a “definitely yes”, “probably yes”, “probably no”, or “definitely no”.

## 3. Results

First, the results of the literature search, screening, and quality assessment are described, followed by a description of the main characteristics of the included studies.

Finally, the results of the studies are considered and divided into four subgroups: emotion regulation assessed by measures of 1. peripheral physiology (ANS), 2. central physiology (CNS; neuroimaging and EEG), 3. biochemical level (cortisol and oxytocin), and 4. nonverbal behavior (facial expressions and vocal characteristics).

### 3.1. Results for Literature Search, Screening, and Quality Assessment

**Figure 2:**
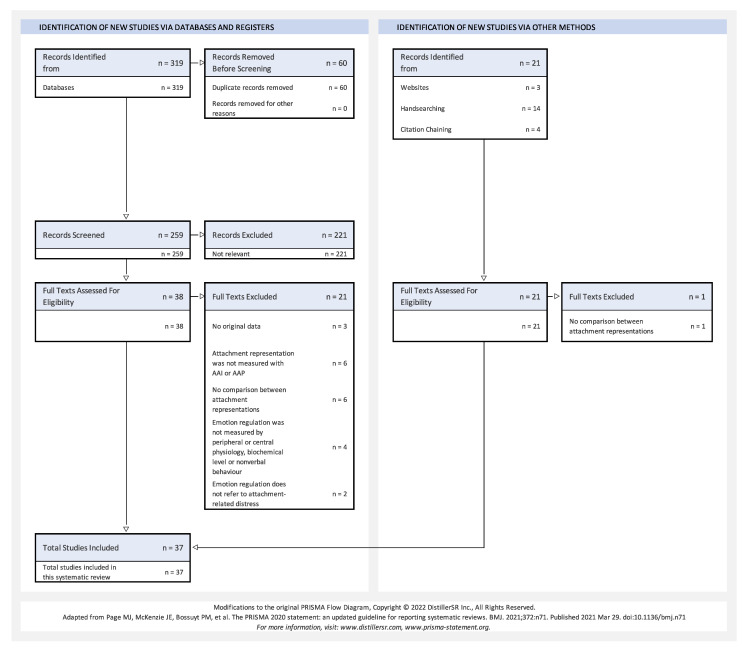
PRISMA flowchart for the conducted search strategy [100].

Figure 2 shows the result of the search strategy and the two screening stages in the form of a PRISMA flowchart. The criteria for excluding particular studies are also indicated there. The duplicates were removed with DistillerSR. The screening was also performed with DistillerSR independently by both authors. The interrater agreement at all screening levels and for the quality assessment of the included studies can be rated as almost perfect [105]. The weighted Cohen’s kappa for screening level 1 (title and abstract) was 0.82, for screening level 2 (full text) 0.98, and for quality assessment 1.0.

The quality assessment of the 37 included studies showed that none of the studies had a high risk of bias. Therefore, all studies remain in the following qualitative analysis.

All measurement methods used within the studies were judged by the authors as reliable and valid (with a few restrictions for some studies that analyzed nonverbal behavior, which is outlined in the appropriate section). The greatest source of potential bias risk was because, in some studies, the sample size was considered to be small (for an overview of the sample sizes, see the tables in each of the sections for the four subgroups). This was especially true for neuroimaging studies, which is common due to the financial costs of this type of research. Similarly, the majority of the CNS study samples consisted of women (10 of 13 studies). This does not allow the results to be generalized to men.

In addition, in general, the dropout rate exceeded 25% in a few cases. This is discussed separately below when presenting the individual studies in the relevant section. Since none of the included studies was judged to have a high bias risk, all studies were included in the following qualitative synthesis.

### 3.2. Main Characteristics of the Included Studies (n = 37)

Before moving into the analysis and synthesis of the results of the included studies (*n* = 37), we briefly summarize the main characteristics of the studies. This is to provide the reader with an overview and, thus, a better understanding of the summarized research of this systematic review.

All included studies together comprise *n* = 2006 subjects, of which 1345 are female (67%). The subjects’ mean age in the included studies ranges from 14.4 years (*SD* = 0.53) to 6500 years (*SD* = 10.00). Twenty-five studies used the AAI to measure attachment representation, and 12 used the AAP.

The earliest included study was published in 1992, and the most recent in 2023. Of the 37 included studies, 17 studies (46%) were published between 2016 and 2023. This indicates an increasing research interest in the topic of this review in recent years. Sixteen studies were conducted in Germany (of which one was simultaneously in Austria), ten in the USA, five in Italy, four in the Netherlands, and one in Portugal.

Aligned with the four objective measurement approaches to emotion regulation described above, the 37 included studies are divided as follows. For the first subgroup, which assessed emotion regulation response via ANS measurements, we identified 11 studies through our search. For subgroup 2 (CNS measurement by neuroimaging or EEG), there are 13 studies. The third subgroup, which is composed of the studies with measurements of emotion regulation response at a biochemical level, there are six studies. Finally, the fourth subgroup (nonverbal measures of emotional response) consists of nine studies. This does not add up to 37, but to 39, because in 2 studies, measurement methods were combined. One study (see Table 4 and Table 7 in the corresponding section) measured emotion regulation response in both peripheral physiology (ANS) and nonverbal behavior. Another study (see Table 5 and Table 6 in the corresponding section) measured the response in both central physiology (CNS) and biochemistry.

### 3.3. Main Results

In the following qualitative synthesis of the included studies, we will divide the analysis and summary of results into four subgroups aligned with the four objective measurement approaches of emotion regulation described in the introductory section.

#### 3.3.1. Subgroup 1: Measurements of Peripheral Physiology (ANS; *n* = 11)

As described in the introduction, ANS measurements reflect either primarily the activity of the sympathetic or parasympathetic branch of the autonomic nervous system or, in addition, also indicate the combined activity of both branches. This classification will guide us here (rather than going through each study in turn). We will first look at the parameters of the sympathetic branch (skin conductance level), then at those of the parasympathetic branch. For the latter, the focus is on heart rate variability (HRV). Finally, we will look at the parameters that reflect a combined activity of both branches of the autonomic nervous system (heart rate and blood pressure).

Table 4 shows an overview of all 11 studies in this ANS subgroup in terms of their main characteristics. Since HRV can be calculated by very different parameters, the exact type of HRV calculation is indicated in brackets after it in the respective study.

**Table 4:** Overview of reviewed studies (in alphabetical order) on attachment-related differences in emotion regulation measured by responses of peripheral physiology (ANS) (*n* = 11).

Author/s (Year)	Country	Sample Size	Population	Gender	Attachment Measures	Measurement of ANS Response	Type of Stressor
Ablow et al. [106] (2013)	USA	53	Healthy	Female	AAI	HR, SCL, HRV (peak-valley)	Infant crying
Balint et al. [107] (2016)	Germany	50	Clinical	Mixed	AAP	HR, BP	Pictures of AAP
Beijersbergen et al. [108] (2008)	Netherlands	152	Healthy	Mixed	AAI	HR, SCL, HRV (RMSSD)	Questions of AAI; conflict task
Dias et al. [109] (2011)	Portugal	47	Clinical	Female	AAI	HR, SCL, HRV (LF/HF ratio)	Questions of AAI
Dozier and Kobak [110] (1992)	USA	50	Healthy	Mixed	AAI	SCL	Questions of AAI
Gander et al. [37] (2022)	Germany, Austria	56	Healthy	Mixed	AAP	HR, HRV (RMSSD)	Pictures of AAP
Holland and Roisman [111] (2010)	USA	230	Healthy	Mixed	AAI	SCL	Couple conflict resolution task
Reijman et al. [112] (2017)	Netherlands	73	Healthy, clinical	Female	AAI	SCL, HRV (RMSSD)	“Comfort paradigm” video clip
Roisman [113] (2007)	USA	160	Healthy	Mixed	AAI	HR, SCL, HRV (HF-HRV)	Couple conflict resolution task
* Roisman et al. [114] (2004)	USA	60	Healthy	Mixed	AAI	HR, SCL	Questions of AAI
Zingaretti et al. [115] (2020)	Italy	59	Healthy	Mixed	AAI	HRV (RMSSD)	Questions of AAI

Legend—AAI (Adult Attachment Interview); AAP (Adult Attachment Projective Picture System); BP (blood pressure); HF (high frequencies); HR (heart rate); HRV (heart rate variability); LF/HF ratio (low/high-frequency ratio); RMSSD (root mean square of successive beat-to-beat interval differences); SCL (skin conductance level). * This study measured the emotion regulation response in both peripheral physiology (ANS) and nonverbal behavior (facial expressions).

##### Studies on Sympathetic Nervous System (Skin Conductance Level)

A total of 8 of the 11 studies measured ANS response via SCL. As mentioned above the skin conductance level indicates primarily an activity of the sympathetic nervous system.

Ablow et al. [106] showed that insecure-dismissing women (compared to securely attached women) responded with a higher sympathetic activity to the attachment-related stressor (an infant cry condition in this study) indicated by statistically significant higher SCL. It is important to emphasize that in this study, only the two attachment representations of secure dismissing and insecure dismissing were compared. Of particular interest was that the insecure-dismissing women, in contrast to the securely attached ones, responded with a significant increase in SCL while watching a video scene in which the child was playing contentedly with his mother. Complementarily, Dozier and Kobak [110] found no significant difference between insecure and secure individuals in SCL reactivity in response to the AAI questions but did find a statistically significant difference between affect regulation strategies. The result was that a deactivation strategy (typical for insecure dismissing) was associated with an increase in SCL during the AAI. This finding was replicated by Roisman et al. [114], who interpreted the increased SCL as a physiological marker of effortful inhibition of behavior. Roisman [113] also demonstrated this correlation. He showed that insecure-dismissing individuals responded with statistically significantly higher SCL during a conflict resolution task with their romantic partners.

In contrast, Dias et al. [109] showed that insecure women showed a statistically significantly higher SCL during the AAI interview than secure women. However, they found no significant correlation between affect regulation strategies of deactivation (typical for insecure-dismissed individuals), respectively, hyperactivation (typical for insecure-preoccupied individuals), and changes in SCL in response to the AAI questions. Similarly, Holland and Roisman [111] showed that generally insecure study participants (but not specifically deactivation) responded with higher SCL compared to secure participants during a conflict resolution task.

Reijman et al. [112] found that individuals with an unresolved attachment representation responded with lower SCL while watching the post-separation reunion between Caregiver and a crying infant in an animated video clip (“comfort paradigm”). The authors of the mentioned study theorize that this, following Kreibig [116], may be a sign of a deactivating response in the manner of non-crying sadness. This would be congruent with the dissociative nature of an unresolved status of mind, according to the authors of this study. The fact that decreased SCL in the subjects with unresolved attachment representation was evident in the reunion scene and not during the viewing of the separation may well be related to the fact that this is contrary to the subjects’ own subjective experience of attachment.

Beijersbergen et al. [108] found no difference in SCL reactivity between attachment classifications of adolescents adopted in early childhood either during the AAI or during a conflict resolution task with their adoptive mother.

##### Studies on Parasympathetic Nervous System (Heart Rate Variability)

A total of 7 of the 11 studies measured ANS response by HRV (4 by RMSSD and one each by peak-valley, HF-HRV, LF/HF ratio), whereas according to Laborde et al. [58], RMSSD, peak-valley, and HF-HRV reflect the vagal tone, i.e., the activity of the parasympathetic branch, the LF/HF ratio should be viewed differently. In their review, they write, “The LF/HF ratio was long considered as representing the sympatho-vagal balance which is the balance between the sympathetic and parasympathetic systems. However, this view has been highly criticized. (…) We strongly recommend researchers to adopt HRV indices that reflect clearly identified physiological systems with a theoretical underpinning such as the indices of vagal tone (i.e., RMSSD, peak-valley, and HF-HRV)”. Because in terms of validity, the implication of the LF/HF ratio for vagal tone is unclear, we will not consider the results of the study by Dias et al. [109] with respect to HRV in the qualitative synthesis.

Ablow et al. [106] observed that women with a secure attachment representation showed a significant decrease in HRV (calculated by peak-valley) in response to the infant cry as an attachment-related stressor, whereas insecure-dismissing women reacted with a significant HRV increase. According to the authors of the mentioned study, the latter can be seen as an attempt to down-regulate the increased arousal (indicated by simultaneously increased SCL) to shift attention away from the attachment-relevant stressor—in contrast to mobilizing behavior that would be directed toward soothing the infant. The decrease in HRV in securely attached women, on the other hand, together with the parallel increased arousal (increased SCL), is a typical indication that the attachment figure is internally aligned with the infant and that behavior is being mobilized to satisfy the infant’s needs and to soothe the infant. For example, studies have shown that the decrease in HRV is also typical in secure mothers in the reunion phase of the Strange Situation and indicates the mother’s attunement to the infant [117].

In contrast, Gander et al. [37] found that HRV (as measured by RMSSD) was statistically significantly higher during the AAP interview (which functioned here as an attachment-related stressor) compared to baseline in secure adolescents than in insecure adolescents. It should be noted that a statistical difference was only found between the securely attached group on the one hand and the two attachment groups, insecure-dismissing and unresolved, on the other. There was no significant difference in HRV between securely attached and insecure-preoccupied adolescents. The authors of the mentioned study positively interpreted the higher HRV of the securely attached adolescents in response to the attachment-related stressor as a better ability to regulate emotions compared to the insecurely attached ones. An important note about this study is that 26.4% of the initial subjects could not be included in the statistical analysis because the physiological measurements were erroneous. This poses a risk of possible bias. Similarly, it should be noted that the main attachment representations of the subjects assessed by the AAP were secure (33.9%) and insecure dismissing (42.9%). This possibly limits the generalizability of the results for the other two attachment groups (insecure-preoccupied and unresolved).

Zingaretti et al. [115] examined the HRV patterns of their subjects according to the Vagal Tank Theory following Laborde et al. [118]. According to the Vagal Tank Theory, not only HRV, as such, is indicative of the quality of emotion regulation but also the change in HRV between the three states of resting, reactivity, and recovery. Using this scheme, the researchers of the mentioned study set up their experiment and showed that subjects with organized attachment representation (with no statistically significant difference between secure and insecure individuals) showed a decrease in HRV (measured by RMSSD) from baseline (resting) to AAI (reactivity) and then returned to the HRV baseline level in the recovery phase. The researchers of this study interpreted this as an adaptive strategy of emotion regulation, which is to respond physiologically to stress when it is present and then recover when the stress is removed. In contrast, subjects with unresolved attachment representation showed an unexpected increase in HRV from baseline to AAI, which remained at elevated levels during the subsequent recovery phase. The increase in HRV during the AAI was interpreted as an active attempt to suppress negative memories and regulate unpleasant emotions. This required an effortful recruitment of resources, which persisted in the recovery phase after the interview.

Beijersbergen et al. [108] found no difference in HRV reactivity (measured by RMSSD) between attachment classifications of adolescents adopted in early childhood, either during the AAI or during a conflict task with their adoptive mother. Likewise, Reijman et al. [112] found no RMSSD differences between attachment classifications while subjects watched an animated video clip (“comfort paradigm”) depicting both a separation and a reunion between caregiver and infant. The same was found by Roisman [113], who also showed no difference in HRV response between attachment classifications when subjects were asked to solve a conflict resolution task with their romantic partner.

##### Studies on combined ANS Activity (Heart Rate and Blood Pressure)

A total of 7 of the 11 studies measured ANS response by heart rate (HR) and/or blood pressure (BP). These two parameters, as mentioned above, reflect a combined activity of the sympathetic and parasympathetic nervous systems.

Beijersbergen et al. [108] found that insecure-dismissing adolescents (all subjects of this study were adopted in infancy before the age of 6 months) showed less reactivity in their heart rate (measured by interbeat intervals; IBI) than securely attached ones during AAI (no differences were found between secure and preoccupied adolescents). Thus, they seemed to be less stressed. The researchers interpreted this, according to their previously defined hypothesis, to imply that the insecure-dismissing adolescents were able to effectively use internal defense strategies to deactivate emotional stress while reflecting on their childhood experiences (in the context of the AAI). However, in a conflict task with their adoptive mother, they showed more IBI reactivity than the securely attached adolescents (but again, no statistically significant difference in IBI reactivity between secure and preoccupied adolescents). So, during the conflict resolution task, the insecure-dismissing adolescents were seemingly more emotionally stressed, which, according to the studies’ authors, could have been triggered by the different demands between AAI and conflict tasks. “It seems impossible to be uninvolved and detached during a direct interaction task with their mother with the goal of reaching consensus in an area of disagreement”, the researchers reason for the difference in their discussion of the results of this study. Thus, in the conflict task with their mother, their defensive strategies no longer worked since they were supposed to be working toward a resolution of the conflict at the same time.

Roisman [113] found that subjects with an insecure-preoccupied attachment representation responded with a higher HR during a conflict resolution task with their romantic partner, which, according to the author, indicates behavioral activation for this attachment classification in response to attachment-related stress.

Balint et al. [107] found a high prevalence of insecure attachment of 88% in a sample of patients with hypertension, which is almost twice as frequent as in a non-clinical sample. However, inconsistent with other research, they found that in this study, secure subjects responded with statistically significant higher systolic blood pressure than individuals with insecure attachment representation to acute attachment-related stress. One explanation may be that this study did not examine normotensive individuals, but patients who suffered from severe hypertension and who were treated with multiple anti-hypertensive agents. Although, apart from this, there were no differences in measured ANS responses (HR and BP) between attachment classification in this study, subjects overall showed an overall increase in heart rate and BP in response to the attachment-related stressor (in this study, the AAP interview).

Ablow et al. [106] found that contrary to their prediction, the average HR of both secure and insecure-dismissing women remained comparable with no statistically significant difference in response to an infant cry condition, which served as an attachment-related stressor in this study. Dias et al. [109] similarly found no difference between securely and insecurely attached subjects in HR response during the AAI interview. Moreover, Gander et al. [37] showed that although HR increased when comparing baseline to AAP interview as an attachment-related stressor among subjects, they also found no significant difference between the attachment groups in the increase in HR. Likewise, Roisman et al. [114] found no statistically significant difference in HR reactivity between attachment classifications during AAI assessment.

#### 3.3.2. Subgroup 2: Measurements of Central Physiology (CNS; *n* = 13)

As already mentioned in the introduction, measurements of central physiology can be divided into two categories: neuroimaging methods and EEG measurements. We will follow this classification and first summarize the studies that have measured brain activity with fMRI. We will then consider the results of EEG research.

Table 5 shows an overview of all 13 studies in this CNS subgroup in terms of their main characteristics.

**Table 5:** Overview of reviewed studies (in alphabetical order) on attachment-related differences in emotion regulation measured by responses of central physiology (CNS) (*n* = 13).

Author/s (Year)	Country	Sample Size	Population	Gender	Attachment Measures	Measurement of CNS Response	Type of Stressor
Buchheim et al. [119] (2016)	Germany	28	Healthy, clinical	Female	AAP	fMRI	Pictures of AAP
Buchheim et al. [40] (2006)	Germany	11	Healthy	Female	AAP	fMRI	Pictures of AAP
Fraedrich et al. [120] (2010)	Germany	16	Healthy	Female	AAP	EEG	Pictures of infant facial expressions
Galynker et al. [121] (2012)	USA	28	Healthy, clinical	Female	AAI	fMRI	Pictures of mother and a female friend
Kim et al. [122] (2014)	USA	42	Healthy	Female	AAI	fMRI	Pictures of infant facial expressions
Kungl et al. [123] (2016)	Germany	40	Healthy	Mixed	AAI	EEG	Emotional memory retrieval
Lenzi et al. [69] (2013)	Italy	23	Healthy	Female	AAI	fMRI	Pictures of infant facial expressions
Leyh et al. [124] (2016)	Germany	25	Healthy	Female	AAI	EEG	Pictures of infant facial expressions
Petrowski et al. [125] (2019)	Germany	38	Healthy	Mixed	AAP	fMRI	Pictures of attachment figures’ faces
Riem et al. [126] (2016)	Netherlands	42	Healthy	Female	AAI	fMRI	Infant crying
Riem et al. [127] (2012)	Netherlands	21	Healthy	Female	AAI	fMRI	Infant crying
* Strathearn et al. [128] (2009)	USA	30	Healthy	Female	AAI	fMRI	Pictures of infant facial expressions
Waller et al. [129] (2015)	Germany	24	Healthy	Male	AAP	EEG	Pictures of smiling infant faces

Legend—AAI (Adult Attachment Interview); AAP (Adult Attachment Projective Picture System); EEG (electroencephalography); fMRI (functional magnetic resonance imaging). * This study measured the emotion regulation response in both central physiology (CNS) and biochemistry (oxytocin).

##### Studies on Brain Activity with fMRI

A total of 9 of the 13 studies measured brain activity via imaging using fMRI. Three of these used pictures of infant facial expressions as attachment-related stressors, and two studies each used pictures of the AAP, pictures of faces with attachment figures, or infant crying as auditory stressors.

Buchheim et al. [119] examined the differences in neural responses to attachment-related stress (activated by picture material of the AAP) between disorganized–unresolved and organized–resolved attachment classifications. A healthy control sample (resolved and unresolved) was compared with borderline patients (all unresolved). They found three main results. First, all unresolved subjects (controls and borderline) showed increased activation of the amygdala in response to the AAP pictures compared to the resolved controls. The authors of this study interpreted amygdala activation as a neural correlate of negative emotional arousal. In accordance with this, as already mentioned in the introduction, Wager et al. [65] showed in a meta-analysis a systematic relationship between avoidance motivation and activity in the amygdala. Second, all controls (resolved and unresolved) responded with increased activation of the rostral cingulate zone (RCZ) compared with the unresolved borderline patients. This indicates a difference in the neural response to attachment-related stress between healthy and borderline individuals. This finding is congruent with the results of a study by Zhai et al. [130] that demonstrated that childhood trauma exposure (which current studies suggest is central to borderline) facilitates neurodevelopmental changes that may impede recruitment of the anterior cingulate cortex (ACC)—particularly the rostral/anterior regions associated with affect processing and regulation—during stressful situations in adolescents with poor inhibitory control. Third, unresolved controls showed increased activation of the right dorsolateral prefrontal cortex (dlPFC). This was not found in resolved controls. According to the authors of this study, research suggests that the right dlPFC fosters, for example, regulatory mechanisms that help individuals cope with extraordinary affordances. On the one hand, this shows that unresolved controls were more emotionally involved via the AAP compared with resolved controls. On the other hand, this indicates that unresolved borderline patients, while emotionally overwhelmed (reflected by an activated amygdala), were neuronally inhibited from initiating adaptive emotion regulation. This could be a neural signature of borderline patients’ inability to exert top-down control under conditions of attachment-related stress, this study’s authors wrote. The pictures of the AAP as attachment-related stressors leading to increased activation of the amygdala in healthy individuals with disorganized–unresolved attachment representation (compared to healthy individuals with organized attachment classification) were also found in a pilot study by Buchheim et al. [40]. During the standardized sequence of pictures of the AAP, according to the theory of this assessment, the attachment system should be increasingly activated. Indeed, in both mentioned studies, the activity of the amygdala successively increased in the unresolved subjects during the AAP picture sequence. The same pattern of increasing activation over time of the AAP picture sequence was also shown in the anterior cingulate cortex (ACC), in this case, for both resolved and unresolved subjects.

In their study, Galynker et al. [121] differentiated between attachment in relation to earlier versus later years by presenting their subjects with either a color photograph of their mother versus a close female friend. As a control condition, they showed a picture of a stranger. Similarly, they analyzed the overlap or uniqueness, respectively, of the neural network associated with insecure attachment versus the neural network linked to depression. Neuronal areas of insecure attachment that overlapped with depression were the orbital and medial prefrontal cortex (PFC) regions, anterior insula, and anterolateral PFC regions. These were also found by Strathearn et al. [128]. In addition, Galynker and colleagues found the ventral putamen, ventral caudate, and medial thalamus to be other activated regions typical for insecure attachment. The latter two were unique to insecure attachment and did not overlap with regions activated in depression. Furthermore, these two regions are, according to the authors of the mentioned study, particularly related to emotionally motivated behavior and memory. Activation of early attachment correlated solely with subcortical activity. Since the stimuli used in this study (in contrast to the two AAP studies mentioned at the beginning of this section) did not represent a per se negative emotional activation, consequently, no activation of the amygdala could be found.

In the just mentioned study by Strathearn et al. [128], mothers were shown pictures of their own infants (smiling versus crying) as attachment-relevant stimuli. Mothers who had a secure attachment showed greater activation of reward regions in the brain (e.g., ventral striatum). Similarly, the hypothalamus/pituitary region associated with oxytocin was more activated. This correlated with an actual higher oxytocin response in contact with the infants at seven months of age. In contrast, insecure-dismissing mothers showed activation of the anterior insula associated with feelings of unfairness, pain, and disgust in response to their infant’s sad facial expressions, for example.

Kim et al. [122] also showed mothers pictures of facial expressions (happy versus sad) of their own versus unfamiliar infants. They demonstrated that mothers with disorganized–unresolved attachment representation had a blunted amygdala response in response to their own infants’ sad (versus happy) facial expressions compared with mothers who were organized–resolved attached. The authors of the mentioned study interpreted this as a possible neural underpinning of a potential disengagement of traumatized mothers from infant stress. For this study, it is necessary to mention that the risk of possible bias is higher because the dropout rate was greater than 25 percent (of 61 mothers originally, 42 participated in this study), and a reason (technical problems) was given for this for only 2 of 19 subjects who were not included in the final study analysis.

Lenzi et al. [69] found that insecure-dismissing nulliparous young adult females showed greater activation of the mirror and limbic areas compared with securely attached ones in response to infant facial expressions and the task of empathizing with them. However, the authors of this study did not interpret this result as an enhanced empathic response but rather as indicative of affective dysregulation driven by a reactivation of infant memories of parental rejection in relation to their own attachment needs. Similarly, this study showed that insecure-dismissing individuals (compared to those securely attached) deactivate the perigenual anterior cingulate cortex (pACC) and medial orbitofrontal cortex (mOFC) in response to stressful infant facial expressions. The authors interpreted this deactivation of fronto-medial regions as a possible neural correlate of attachment avoidance behavior typical of individuals with an insecure-dismissive attachment representation.

Petrowski et al. [125] found no difference in activated brain regions between securely and insecurely attached individuals in response to pictures of faces of attachment figures (versus unfamiliar individuals) but between individuals with organized–resolved and disorganized–unresolved attachment representations. Securely attached healthy adults showed activation in areas of the neural social judgment network (e.g., inferior parietal lobe/superior temporal gyrus). This brain network is important for inferring the emotional and cognitive mental states of others. In contrast, individuals with disorganized attachment showed deactivation in these areas, which, according to the authors of the mentioned study, might reflect an ineffective form of stress and emotion regulation. This finding is supported by a meta-analysis [131] that found a statistically significant correlation between mentalizing ability and secure attachment.

Riem et al. [127] showed that insecurely attached individuals responded with heightened amygdala activation in response to an attachment-relevant auditory stress stimulus, specifically infant crying, compared with securely attached individuals. In another study by Riem et al. [126], this result could be replicated. Moreover, the researchers demonstrated that the administration of intranasal oxytocin decreased amygdala reactivity in insecurely attached individuals during exposure to infant crying. This is as well indicative of the central link between oxytocin and a secure attachment representation.

##### Studies on Brain Activity with EEG

In 4 of the 13 studies, researchers used EEG to measure brain activity. In three of them, pictures of children’s facial expressions were used as attachment-related stressors and, in one, emotional memory retrieval.

The link between oxytocin and secure attachment just described was also found in an EEG study by Waller et al. [129]. They found that intranasal administration of oxytocin moderated event-related potentials (ERPs) in fathers in response to attachment-relevant faces (a picture of their own infant versus a familiar and an unfamiliar infant; all pictures with facial expressions with positive valence) in relation to N250 and N300 components. These ERPs, whose amplitude was, in general, larger in response to pictures of fathers’ own infants, correlated with activation of face representations in memory and attribution of emotional valence, respectively. Oxytocin administration affected the abolishment of the differences in ERPs between the different picture stimuli. This effect was statistically significantly larger for secure than for insecure fathers, which is indicative that the effect of oxytocin is influenced by the characteristics of the attachment representation.

Fraedrich et al. [120] found that mothers with secure attachment representations differed from insecure-dismissing mothers in their responses to infants’ presented facial expressions on the EEG in their ERPs. Insecure-dismissing mothers showed greater N170 amplitudes in response to infant facial expressions, regardless of their valence. The authors of this study interpreted this to indicate that for insecure-dismissing mothers—because they had to activate more processing resources than secure mothers—configural encoding of faces was harder. This fits with the assumptions and empirical findings of attachment theory, which emphasize a link from secure attachment to social orientation and competence. In addition, secure mothers responded with a larger N200 amplitude to all face stimuli compared with the insecure-dismissing ones. According to the authors, this can be understood as an indication that secure mothers pay more attention to faces and are, thus, more able to perceive the emotional facial expressions of infants, which is an essential aspect of maternal sensitivity. Similarly, in response to flower stimuli (as a control condition), the secure mothers showed a larger amplitude in the attention-related P300 component compared to faces as the standard stimulus. According to the authors, this is indicative of a perceptual bias towards social stimuli in secure attachment subjects. Regarding frontal asymmetry, although the results did not reach statistical significance (probably due to the small sample size), the insecure-dismissing mothers, nevertheless, showed greater activity in the right frontal lobe, which research indicates characterizes avoidance motivation.

Leyh et al. [124] replicated and further differentiated the findings of the study just mentioned. By showing the mothers in their study pictures of infants who were the same age as the infants of the mothers, they found that insecurely attached mothers (preoccupied and dismissing were grouped together due to small size; the response patterns between the insecure subgroups showed no statistically significant difference) showed larger N170 amplitudes than secure mothers in response to facial expressions of unpleasant emotion. This emphasized the role of negative emotional facial expressions in the larger demand for the configural encoding of nonverbal emotional signals in insecure mothers. In addition, the secure mothers again showed a larger P300 amplitude compared to the insecure mothers, which, as mentioned above, is indicative that attention increases for secure mothers in emotionally relevant contexts. In the insecure mothers, not only was the P300 amplitude smaller than in the secure subjects but at the same time the P300 latency was elevated. This suggests that insecure mothers respond with slowed-down processing of emotional facial cues in the context of expressed infant emotions.

Kungl et al. [123] found that insecure-dismissing individuals showed elevated activity in the right frontal brain at rest. This was interpreted by the authors, in congruence with attachment theory, as a disposition of individuals with insecure-dismissing attachment representations toward withdrawal strategies. Retrieval of happy, sad, and angry emotional memories from adolescence (which served as an attachment-related stressor in this study) did not affect frontal asymmetry. However, both subgroups of insecure attachment showed increased right-sided parietal activity during the emotional task compared to the resting state, which was interpreted as an indicator of challenging the emotion regulation ability of insecurely attached during the retrieval of emotional memories.

#### 3.3.3. Subgroup 3: Measurements of Biochemistry (*n* = 6)

In the studies summarized subsequently, that examined the emotion-regulatory response to attachment-related stressors and that we identified through our search strategy, all but one (which included DHEA) examined the effect on either oxytocin or cortisol or the effect on both. We will first consider the studies that included both in their analysis, then summarize the oxytocin studies, and conclude with the studies regarding cortisol.

Table 6 shows an overview of all six studies in this biochemistry subgroup in terms of their main characteristics.

**Table 6:** Overview of reviewed studies (in alphabetical order) on attachment-related differences in emotion regulation measured by responses of biochemistry (*n* = 6).

Author/s (Year)	Country	Sample Size	Population	Gender	Attachment Measures	Measurement of Biochemistry	Type of Stressor
Jobst et al. [132] (2016)	Germany	41	Clinical	Female	AAP	Cortisol, Oxytocin	Social exclusion (cyberball game)
Karabatsiakis et al. [133] (2022)	Germany	26	Healthy	Male	AAP	Cortisol, DHEA, Oxytocin	Pictures of AAP
Krause et al. [134] (2016)	Germany	44	Healthy	Female	AAP	Cortisol, Oxytocin	Pictures of AAP
Petrowski et al. [135] (2017)	Germany	98	Healthy	Mixed	AAP	Cortisol	Trier Social Stress Test
Rifkin-Graboi [136] (2008)	USA	37	Healthy	Male	AAI	Cortisol	Simulated interpersonal stress
* Strathearn et al. [128] (2009)	USA	30	Healthy	Female	AAI	Oxytocin	Pictures of smiling infant faces

Legend—AAI (Adult Attachment Interview); AAP (Adult Attachment Projective Picture System); DHEA (dehydroepiandrosterone). * This study measured the emotion regulation response in both biochemistry and central physiology (fMRI).

Jobst et al. [132] reported no statistically significant difference comparing borderline patients to healthy individuals in their response to social exclusion in a cyberball game as an attachment-related stressor considering different attachment classifications. Only a statistically significant lower baseline oxytocin plasma level was found in disorganized–unresolved attachment compared to an organized attachment within the borderline group.

Krause et al. [134] found that AAP picture stimuli, as attachment-related stressors, resulted in increases in oxytocin and decreases in cortisol levels in breastfeeding mothers compared with baseline. This result did not confirm the hypothesis that secure mothers show a greater increase in oxytocin in response to attachment-related stress. Similarly, no statistically significant difference in cortisol reactivity was found between the four attachment subgroups. However, mothers with insecure attachments tended to respond with a greater oxytocin increase in response to the AAP. Additionally, mothers with secure attachments tended to have a larger decrease in cortisol. It should be noted for this study that the subjects were breastfeeding mothers, whose oxytocin system is more sensitive. Similarly, the sample sizes for the individual attachment subgroups were small. In addition, the dropout rate was more than 25 percent (44 of 67 mothers were finally included in this study’s analysis). What should be emphasized here is that a plausible reason was given for the dropout of each (e.g., insufficient blood samples or technical problems). Nevertheless, these circumstances limit the generalizability of the results.

Karabatsiakis et al. [133] attempted to replicate the results of the study just mentioned with new fathers. In addition, the change in DHEA was investigated. Although the men with disorganized–unresolved attachment representation (compared with organized attachment) showed higher basal cortisol levels (before the AAP), no difference was found for either cortisol or DHEA in response to the AAP among the subjects. On average, subjects’ oxytocin levels increased in response to the AAP stimuli, which was statistically significantly more pronounced in the unresolved fathers.

Strathearn et al. [128] found, in the study already mentioned in the CNS subgroup’s results section, congruent with their predictions that first-time new mothers with secure attachment not only showed activation of oxytocin-relevant brain regions in response to infant cues but, in addition, compared with insecure-dismissing mothers, responded with increased oxytocin release during interactions with their infants.

Petrowski et al. [135] demonstrated that individuals with disorganized–unresolved attachment representation compared with organized attachment showed statistically significant prolonged cortisol recovery after a social stressor. In contrast, they did not find an attachment-related difference in cortisol reactivity as an immediate response during stress exposure. The researchers used the Trier Social Stress Test (TSST) as an experimental stressor. Even if this is not an entirely comparable attachment-related stressor, the TSST can still be interpreted as such. This is because the situation of personal evaluation, the rejection experienced through negative evaluation, and the time and evaluation pressure are likely to threaten the attachment system.

Rifkin-Graboi [136] found no difference between insecurely attached and securely attached individuals in basal cortisol levels. However, a statistically significant difference was found in cortisol reactivity in response to attachment-related stress simulated in the laboratory. After subjects visualized and responded to hypothetical situations concerning loss, separation, and abandonment, insecure-dismissing subjects (the number of insecure-preoccupied subjects was too small to include in the analysis) showed higher cortisol levels compared with secure ones. It is important to note that the dropout rate of this study was around 50 percent (only 37 of the initial 73 male students participated finally in this study). Reasons were not given for all excluded subjects; therefore, the bias risk in this study is of a larger magnitude.

#### 3.3.4. Subgroup 4: Measurements of Nonverbal Behavior (*n* = 9)

The subsequently summarized studies can be divided into research that used (1) the Facial Action Coding System (FACS) to code facial behavior (four of the nine included studies), (2) captured facial expression movement with a Facial EMG (two studies), (3) conducted a prosodic analysis using PRAAT speech software (two of the nine studies), or (4) measured interaction behavior with the Specific Affect Coding System (SPAFF) (one study). For comprehensibility, we will analyze the studies in exactly this listed order and categorization.

It should be noted for the studies which used the Facial Action Coding System (FACS) for coding nonverbal movement behavior, that each of these studies has certain limitations (e.g., in the facial coding per se or in the sample size) that result in a limitation of the results regarding the analysis of attachment-related facial behavior patterns. An overarching reason for the limitations identified is that manual coding of facial expressions is very time consuming. Therefore, this is usually conducted only with a small sample size, or the scope of facial coding as such is limited from the beginning regarding the Action Units (AUs) to be considered. The specific limitations of the particular studies and their consequences are discussed below for each study separately.

Table 7 shows an overview of all nine studies in this nonverbal behavior subgroup in terms of their main characteristics, listed in alphabetical order of authors.

**Table 7:** Overview of reviewed studies (in alphabetical order) on attachment-related differences in emotion regulation measured by responses of nonverbal behavior (*n* = 9).

Author/s (Year)	Country	Sample Size	Population	Gender	Attachment Measures	Measurement of Nonverbal Behavior	Type of Stressor
Altmann et al. [137] (2021)	Germany	95	Healthy, clinical	Mixed	AAI	FACS, MEA	Questions of AAI
Babcock et al. [138] (2000)	USA	36	Healthy	Male	AAI	SPAFF	Couple conflict resolution task
Buchheim et al. [139] (2007)	Germany	39	Healthy, clinical	Female	AAP	EmFACS	Pictures of AAP
Buchheim and Benecke [140] (2007)	Germany	27	Healthy, clinical	Female	AAI	EmFACS	Questions of AAI
Fossataro et al. [141] (2023)	Italy	26	Healthy	Mixed	AAI	Facial EMG (eye-blink reflex)	Pictures of AAP; threat of peripersonal space
* Roisman et al. [114] (2004)	USA	60	Healthy	Mixed	AAI	FACS	Questions of AAI
Spangler and Zimmermann [102] (1999)	Germany	35	Healthy	Mixed	AAI	Facial EMG (smile, frown, eye-blink reflex)	Attachment-related film scenes
Spinelli et al. [142] (2022)	Italy	67	Healthy	Female	AAI	Prosodic analysis with speech software	Questions of AAI
Spinelli et al. [143] (2019)	Italy	77	Healthy	Female	AAI	Prosodic analysis with speech software	Questions of AAI

Legend—AAI (Adult Attachment Interview); AAP (Adult Attachment Projective Picture System); EmFACS (Emotion Facial Action Coding System); Facial EMG (facial electromyography); FACS (Facial Action Coding System); MEA (Motion Energy Analysis); SPAFF (Specific Affect Coding System). * This study measured the emotion regulation response in both nonverbal behavior and peripheral physiology (SCL; skin conductance level).

##### Studies on Facial Behavior Using Facial Action Coding System

Altmann et al. [137] compared the nonverbal patterns of individuals with secure, insecure-dismissing, and insecure-preoccupied attachment representations. They analyzed both the frequency and complexity of overall nonverbal movement behavior and the frequency of specific emotional facial expressions (happiness, sadness, and contempt). They controlled the results for any interactions with mental disorders (anxiety disorder and anxiety disorder with comorbid depression). They found that, consistent with their hypothesis, insecure-dismissing individuals overall engaged in nonverbal movement behaviors that were statistically significantly less frequent and less complex than secure and insecure-preoccupied attached individuals. In contrast to their expectations, however, no attachment-related difference was found in the frequency of occurrence of the three coded emotional facial expressions happiness, sadness, and contempt during the first five questions of the AAI examined in this study. However, as expected, an interaction of attachment and the presence of a mental disorder in relation to the observed facial behavior was shown. In the subgroup of patients with anxiety disorder and comorbid major depression, securely attached individuals showed more facial expressions of happiness and more facial movement overall than insecure-dismissing patients. It is important to note as a limitation in coding facial behavior for this study that (a) only a limited set of facial action units, which were not specified, were coded, and (b) this coding was carried out in an automated way via software called OpenFace, resulting in a limitation in validity compared to coding facial behavior by certified FACS coders.

Buchheim and Benecke [140] also found an interaction between attachment and mental disorders regarding different patterns in facial behavior. They showed in a pilot study that securely attached healthy individuals expressed happiness in their facial expressions during the first six questions of the AAI statistically significantly more often than insecurely attached healthy individuals as well as insecurely attached patients with anxiety disorder but also more often than securely attached anxiety patients. Facial expressions of happiness seemed to be associated with mental health and attachment security. Although the difference was not statistically significant, insecurely attached patients with anxiety disorder nevertheless tended to express social smiles more frequently, whereas they reported in a mainly incoherent way, e.g., about previous experiences of separation. The authors interpreted this as an indication of self-regulatory defense. It should be noted for this study that the authors limit the validity of the results presented regarding facial behavior. They rationale this by the fact that, due to the very high facial coding effort, only individual particularly stressful questions were selected from the AAI and facially coded. Furthermore, the sample size was small, which resulted in very low subgroup numbers, especially for the calculations of interactions. The authors argue that this fact did not allow the use of a formation of subgroups to examine the four attachment patterns with the respective psychopathology (subgroups of anxiety disorders).

Roisman et al. [114] analyzed emotional facial expressions as a function of individuals’ attachment representations not only during the initial questions of the AAI but throughout the entire Adult Attachment Interview. They found that insecure-preoccupied individuals were statistically significantly more frequently to express unpleasant emotions in their facial expressions. They also found that securely attached individuals had a high level of coherence between the valence of emotions expressed in facial expressions (positive versus negative) and the subjectively rated valence of emotional experience when reporting positive and negative childhood experiences, respectively. For secure and insecure-dismissing individuals, no significant differences in facial behavior were found in this study in relation to the frequency of showing positive versus negative emotional facial expressions. A clear limitation of this study in relation to the coding of facial behavior, however, is that in order to save time, facial coding via FACS was limited to only two broad categories (positive versus negative valence of facial expressions). Positivity, however, was measured, for example, only by raising the corners of the mouth (Action Unit 12). Here, the coding and subsequent analysis did not distinguish between social smiles (AU12) and expressions of happiness (AU6 + 12). Overall, the enumerated limitations impair a differentiated consideration and analysis of attachment-related facial patterns.

Buchheim et al. [139] showed in a study with female borderline patients (compared to a healthy female control group) that in response to attachment-related stress (induced by the picture stimuli of the AAP), unresolved borderline patients showed statistically significantly more disgust in their facial expressions. Unresolved healthy women in the control group facially displayed a tendency for more frequent expressions of contempt. However, this result should be interpreted with caution because only five women were assigned to this subgroup. The authors of this study interpreted the facial emotional expression of contempt as an affective attempt at distancing. However, it should be noted, for this study, the small sample size of 15 borderline patients and 24 healthy women in the control group. This fact did not allow them to form subgroups in order to examine the four attachment patterns differentiated in relation to their facial behavior patterns and their interaction with borderline personality disorder.

##### Studies on Facial Behavior Using Facial EMG

Spangler and Zimmermann [102] investigated attachment-related differences in facial behavior using Facial EMG. While the subjects watched attachment-related film scenes, the activity of two facial muscles (zygomaticus major and corrugator supercilii) was recorded. These two muscles were the frown and smile muscles, respectively. In addition, the eye-blink reflex was measured using EMG of the orbicularis oculi muscle. No statistically significant difference was found regarding attachment-related differences in the startle response measured by an eye blink. However, the authors of this study acknowledge that this may be due to methodological weaknesses of this study when viewed from this perspective, as no pictures were used as stimuli here but rather more complex film scenes, which have more variance in emotional valence than pictures due to their dynamic quality. However, the researchers found that insecure-dismissing individuals showed an overall statistically significant lower activation of the frown muscle than secure or insecure-preoccupied subjects. Thus, the facial behavior of insecure-dismissing subjects was more unmoved. Furthermore, the coherence between the negative emotional valence of the film scenes and the activation of the frown muscle was statistically significantly larger in the secure subjects than in both insecure subgroups. No statistically significant attachment-related differences were found for the coherence between the positive emotional valence of the film scenes and the activation of the smile muscle. This may be due to the fact that the zygomaticus major alone cannot distinguish between a social smile (also in the form of a masking smile) and the expression of happiness. For this study, it should be noted that the sample size of the attachment subgroups was very small (e.g., only six individuals were classified as insecure-preoccupied), which limits the generalizability of the results.

Fossataro et al. [141] studied the hand-blink reflex (HBR) as a subcortical defense response of the peripersonal space (PPS), the space surrounding the body. Responding appropriately to threats of the PPS and defending this space when necessary is crucial for survival. In their study, the researchers analyzed attachment-related differences in the defense response of the PPS. The researchers elicited the HBR via transcutaneous electrical stimulation of the right median nerve at the wrist with a bipolar surface electrode. While facial EMG was used to measure the activity of the orbicularis oculi muscle, the intensity of the HBR was measured in relation to three hand positions: far, near, and ultra near. This experimental procedure was conducted twice, each time with a baseline measurement at the beginning: (1) without activation of the attachment system and (2) with a previous activation of the attachment system using the AAP as a stimulus. A statistically significant difference was found between individuals with organized and disorganized attachment representation (due to the small sample size, further differentiation of organized attachment patterns was not possible). The individuals with an organized attachment responded with a linear enhancement of the HBR. It increased with the nearer the hand was to the face. In contrast, the disorganized individuals responded with an abrupt increase in HBR in the ultra-near position compared with the far and near positions. Thus, it seems that the defensive boundaries of the PPS in disorganized attachment classification are limited to the area directly around the face instead of being gradual in shape depending on the proximity of the hand to the face. In addition, the following inverse defensive response pattern was found between the two attachment groups in relation to an activated or “resting” attachment system: organized individuals showed a stronger HBR in the baseline condition and a weaker one in the post-AAP condition (corresponding to an activated attachment system). Thus, the activated attachment system seemed to serve as a coping resource for them. In contrast, disorganized individuals responded with a statistically significantly increased HBR in the post-AAP condition compared with the baseline condition. Thus, the activation of the attachment by the AAP system seemed to alert them emotionally, such that they showed increased PPS defensive responses.

##### Studies on Vocal Prosodic Patterns

Spinelli et al. [143] compared secure with insecure-dismissive individuals in relation to vocal prosodic patterns in response to attachment-related stress evoked by AAI questions in this study. Although the two attachment groups did not generally differ in their pitch, pitch variability, or speech rate in response to the AAI questions, a difference was found in the degree of coherence between these vocal features and the valence of verbal descriptors. Namely, the researchers analyzed at the vocal content level the subjects’ responses to the third and fourth AAI questions. Here, subjects were asked to define five adjectives describing their relationship with their mother and father, respectively, in early childhood. These adjective attributions correspond to the verbal descriptors mentioned above. A statistically significant difference was found for the interaction between the valence of verbal descriptors and pitch variability as well as speech rate. In secure subjects, the valence of verbal descriptors matched the prosody expressed through the voice. Insecure-dismissing subjects, on the other hand, described negative experiences, which are typically cognitively downplayed and normalized by this attachment group in the AAI narrative, with voice prosody that characterized high emotional arousal (fast speech rate and high pitch variability). Conversely, in insecure-dismissing subjects, positive verbal descriptors were characterized by vocal prosody typical for neutral or low-intensity emotions (pitch variability was very low, and speech rate was the same as that found in secure subjects for neutral descriptors). The authors interpreted these results in that the internal working model of attachment strongly influences the degree of coherence between vocal affective and verbal features of emotionally related narratives during the AAI.

In another study, Spinelli et al. [142] examined coherence between vocal prosodic features and verbal content also during AAI, but here, for AAI questions 7, 8, and 9, which relate to experiences of first separation from parents, parental rejection, and possible parental threat. The result was consistent with that of the previously described study. First, responses to AAI questions showed no difference between secure and insecure-dismissing individuals regarding the mean scores of the studied vocal characteristics. Therefore, according to the authors, it can be assumed that the degree of vocally assessed emotional activation by the AAI is not significantly influenced by the attachment pattern. Second, the researchers again found that insecure-dismissing subjects compared to secure individuals showed incoherence between verbal content valence and vocal prosodic characteristics in this study while reporting separation as well as experiences of parental rejection and threat. In addition, as a new finding, they found that the difference in the verbal-prosodic association between attachment groups was related to the experience of parental rejection. The more parental rejection they had experienced, the more secure and insecure-dismissing subjects differed in the degree of verbal-prosodic coherence. According to the authors, in relation to emotion regulation, this result is indicative of the tendency of individuals with a secure attachment to emotionally reprocess and coherently reorganize even and especially negative experiences in order to maintain an emotional balance.

##### Studies on Interactional Nonverbal Behavior

Babcock et al. [138] used the Specific Affect Coding System (SPAFF) to analyze the interactional behavior of domestically violent and maritally stressed but nonviolent husbands toward their wives in an argument in the laboratory. The first finding was that violent husbands (74%) were more likely to be classified in one of the two organized–insecure subgroups or the disorganized–unresolved subgroup than distressed–nonviolent husbands (38%). Of the latter, not a single subject was classified as unresolved. The researchers found the following statistically significant differences between attachment groups in a marital conflict resolution task conducted in the laboratory: secure husbands behaved in a less domineering manner toward their wives than insecure-dismissing and insecure-preoccupied husbands. Insecure-dismissing individuals showed more stonewalling than the other two attachment groups, congruent with attachment theory notions. Stonewalling displays an avoidance motivation and signals to the other person an unwillingness to engage in communicative exchange. Similarly, insecure-dismissing husbands expressed the most contempt toward their wives, whereas securely attached individuals displayed the least contempt. Insecure-preoccupied husbands fell between the other two attachment groups in relation to displaying contempt. Stonewalling and contempt, according to studies by Gottman and Levenson [144], are two communicative-emotional signals that are particularly destructive to marital stability and relationship satisfaction. Secure husbands, contrary to prediction, did not display more positive signals (e.g., humor, affection, and validation) in communications with their wives but did display more defensiveness. However, this could be explained by the fact that the sample selection was limited in that they were either domestically violent or stressed husbands. Likewise, it should be noted that, according to the authors, defensiveness is considered a relatively low-level negative behavior in research. There was also no statistically significant difference between attachment groups in relation to displaying belligerence or anger.

## 4. Discussion

Overall, the results of this systematic review demonstrate that there is a significantly relevant correlation between an individual’s attachment representation and emotion regulation. The previously described results are subsequently interpreted. The interpretation of the results is then followed by a discussion of practical implications for psychotherapy and coaching. This section is concluded with a discussion of the bias risk of the included studies, as well as ideas for future research that can be found in light of the current results.

### 4.1. Interpretation of Results

Subsequently, the previously described results are first interpreted in the categorization of the four subgroups of the measurement of emotion regulation. Finally, these results are synthesized in their interpretation to form an overall conclusion. Additionally, specific ideas for future research are formulated here for each of the emotion measurement subgroups.

#### 4.1.1. Subgroup 1: Measurements of Peripheral Physiology (ANS; *n* = 11)

A total of 8 of the 11 ANS studies measured the extent of sympathetic activation during the response to attachment-related stressors using SCL. Four of these studies showed higher sympathetic activation for an insecure-dismissing attachment representation compared with a secure attachment classification. It is interesting to note here that insecure-dismissing individuals show heightened sympathetic activation in response to attachment-related but neutral stimuli (e.g., watching a video sequence showing an infant playing contentedly with its mother) on the one hand, and during AAI on the other, where it is typical for this attachment classification to downplay negative childhood experiences. In this sense, this response pattern can be considered congruent with attachment theory as effortful inhibition of the need for attachment. Similarly, it supports the notion, also congruent with attachment theory, that individuals with an insecure-dismissing attachment representation tend to show incoherence between subjective experience and physiological stress response. Two other studies did not demonstrate this correlation of higher sympathetic activation in response to attachment-related stressors specifically for insecure dismissing, but only generally for insecure attachment representation. In contrast, one study found no attachment-related correlation regarding SCL response. For the comparison of SCL response between individuals with unresolved attachment representation versus organized attachment, only one study was identified. This showed that unresolved individuals responded with decreasing sympathetic activation to a reunion video scene between caregiver and infant. This was interpreted by the authors as an indication of deactivating sadness and, thus, according to the authors of this study, is a sign congruent with the dissociative nature of the unresolved status of the mind. However, since only one study is available here so far, further studies are needed to shed further light on this relationship.

Thus, for the SCL studies, in summary, the majority of studies, especially for insecure-dismissive individuals, are indicative of heightened sympathetic activation in response to attachment-related stressors, but further research is still needed to further confirm this relationship due to inconsistencies in some other studies. Similarly, further studies are needed to examine the difference between organized and disorganized attachment representation. The latter is relevant to practice because disorganized attachment classification, in particular, correlates with psychopathological diagnoses.

A total of 7 of the 11 studies measured attachment-related differences in parasympathetic activation in response to attachment-related stressors using HRV. Three of these studies found no correlation between an individual’s attachment representation and HRV response. The remaining studies found partly contradictory response patterns. For example, one study showed that secure compared to insecure individuals responded with a decrease in HRV, whereas another study demonstrated that HRV increased in secure attachment compared with insecure in response to attachment-related stressors. By some authors, the increase in HRV was taken as a positive indication of adaptive emotion regulation, and by others, it was taken as a maladaptive attempt to suppress unpleasant emotions. Thus, further research is needed to make clear statements regarding the HRV response in relation to attachment-related differences in response to attachment-related stressors. Beyond examining attachment-related HRV response differences, a consistent scheme for measuring and interpreting HRV responses would also be needed here. To address the latter, Laborde et al. [58] and Laborde et al. [118] already made suggestions.

A total of 7 of the 11 studies measured ANS response via HR and/or BP. Two indicators reflect combined sympathetic and parasympathetic nervous system activity. In relation to attachment-related differences regarding BP reactivity, the studies as a whole did not find any correlation. However, a generally higher prevalence of hypertension in individuals with insecure attachment classification was found. Five studies found no difference in HR responses between attachment classifications. One of these studies examined emotional response to infant cries, and the other four during an attachment interview using the AAI or AAP. Another study examined the response to two different types of attachment-related stressors, one involving questions from the AAI and the other a conflict resolution task. The authors of this study found that while insecure-dismissing adolescents did not show a significant difference in HR response during the AAI, during a conflict resolution task with their mother, HR increased, indicating that they were more stressed here. This shows how important it may be to compare different types of attachment-related stressors, as the demands may be different and, as a result, evoke different emotional responses. In contrast, another study demonstrated an increase in HR during a conflict resolution task (here with the romantic partner) only for insecure-preoccupied individuals but not for the insecure-dismissing attachment classification.

Thus, regarding HR reactivity, current research either does not find a correlation or has inconsistent results. Therefore, further research is needed to come to clear conclusions. However, the research on ANS responses seems to be more promising for SCL and HRV, especially since these two indicators can be clearly assigned to sympathetic and parasympathetic activation, respectively.

In summary, for the investigation of attachment-related differences in ANS response to attachment-related stressors, typical response patterns were found primarily for insecure-dismissing individuals. Their response pattern to attachment-related stressors seems to be one of higher sympathetic activation. This is consistent with attachment theory considerations. Surprisingly, no clear ANS patterns in the sense of increased stress activation have been found so far for insecure-preoccupied individuals.

#### 4.1.2. Subgroup 2: Measurements of Central Physiology (CNS; *n* = 13)

A total of 9 of the 13 studies used neuroimaging to examine attachment-related differences in emotional response to attachment-related stress. These studies found five outcomes in particular. First, two studies showed that a disorganized–unresolved attachment representation compared to an organized–resolved attachment correlated with increased amygdala activation in response to attachment-related stress. This can be interpreted as an indication of an activated avoidance motivation. Here, as well, the type of attachment-related stressor seems to play a role. If the stressor did not consist of abstract stimuli not directly related to the specific individual (as is the case during the AAP) but of specific stressors relevant to the individual (e.g., pictures of one’s own infants with sad facial expressions), another study showed that the amygdala was not more activated but blunted in disorganized–unresolved attachment. Considering the context, this can also be interpreted as an indication of an avoidance motivation, here in the sense of a possible disengagement of the unresolved mothers from the stress expression of their infants. This was also indicated in another study, which showed that a disorganized–unresolved attachment representation correlates with a deactivation of the neural social judgment network and is thus seemingly associated with a diminished mentalizing capacity. In summary, then, individuals with a disorganized–unresolved attachment classification showed increased avoidance motivation in response to attachment-related stressors compared to organized–resolved individuals. Second, the interaction between attachment representation and psychopathological diagnosis also played a role. In one study, both unresolved and resolved healthy subjects showed increased activation of emotion-regulating neural networks (e.g., ACC) compared to unresolved borderline patients. This indicates that it is important to include psychopathological screening in studies investigating attachment-related differences in emotion regulation in order to account for such interactions in this way. Third, mothers with secure attachment classification showed increased activation of neural networks associated with oxytocin in response to attachment-related stimuli. This can be interpreted as indicative of adaptive emotion regulation and activated attachment approach motivation. Fourth, within organized attachment for insecure-dismissing individuals, a response pattern of increased activation of an attachment avoidance motivation was found. In addition, two studies found heightened amygdala activation in response to attachment-related stress for insecure individuals (compared to secure attachment). The researchers also found that intranasal oxytocin administration soothed the amygdala in insecurely attached subjects. This, fifth, together with the result just described (see third finding), is indicative of the correlation of the oxytocin system with secure attachment.

As an overall result of the neuroimaging studies, consistent with notions of attachment theory, it can be summarized that insecure attachment, but especially disorganized–unresolved attachment, is associated with increased avoidance motivation and decreased emotion regulation ability in response to attachment-related stress.

The four EEG studies essentially indicate that securely attached persons pay more attention to infant facial expressions and process them cognitively more easily. Since studies have shown that stress potentially reduces empathy (i.e., makes the processing of emotional facial expressions more difficult) (e.g., [145]), this can be seen as indicative of more adaptive emotion regulation of secure individuals. For individuals with an insecure attachment representation, this is indicative of diminished emotion regulation in response to attachment-related stressors. Regarding frontal asymmetry, the studies indicated that insecure-dismissing individuals have elevated activity in the right frontal brain. This is consistent with attachment theory and is indicative of the tendency toward distancing strategies of insecure-dismissing individuals. However, further studies are needed to confirm this correlation.

#### 4.1.3. Subgroup 3: Measurements of Biochemistry (*n* = 6)

Five of the six studies examined attachment-related differences regarding cortisol reactivity in response to attachment-related stress. Four of these studies found no statistically significant difference in cortisol levels in the immediate response to stress. Only one study found that insecure-dismissing individuals (insecure-preoccupied subjects were not included in the statistical analysis due to small numbers) showed higher cortisol release in response to attachment-related stressors. In addition, one study found that individuals with disorganized–unresolved attachment representation had a prolonged phase of cortisol recovery in the period following the stressful stimulus. Although further studies at the biochemical level are needed to further confirm this relationship, these findings suggest that insecure attachment (including unresolved attachment classification) is correlated with diminished emotion regulation ability. However, it should be emphasized that this correlation was found in only two studies.

Four of the six studies analyzed the differences between attachment groups in oxytocin response, whereas three of the four studies did not confirm the attachment theory hypothesis that more oxytocin is released in response to attachment-related stimuli in individuals with secure attachment than in individuals with insecure attachment classification. One study even found that in disorganized–unresolved attachment, oxytocin levels increased more than in persons with resolved attachment representation. A possible hypothesis here is that oxytocin acts as a kind of stress buffer and therefore increased. Indeed, in this study, unresolved subjects had higher basal cortisol levels before stress confrontation. Only one study showed that, consistent with attachment theory, secure mothers interacted with their infants with higher oxytocin release than insecure-dismissing mothers. Again, more studies are needed to further investigate the correlation between attachment classification and oxytocin reactivity.

#### 4.1.4. Subgroup 4: Measurements of Nonverbal Behavior (*n* = 9)

Four of the nine studies that examined facial behavior using the Facial Action Coding System (FACS) found preliminary indications of attachment-related differences in facial expressive behavior, but these require further clarification and research. One study did not find attachment-related differences in facial expressions of happiness, sadness, and contempt during the first five questions of the AAI. However, when the psychopathological diagnosis was included in the analysis, it was found that individuals with anxiety disorder and comorbid depression who had a secure attachment representation expressed more happiness in facial expressions and, likewise, displayed more facial movement than insecure-dismissing individuals. This again shows that it is probably important to consider the interaction between attachment classification and psychopathology in the analysis of attachment-related differences in emotion regulation. Regardless of psychopathological diagnosis, it was equally found that insecure-dismissing individuals were generally less moved in nonverbal behavior than secure and insecure-preoccupied individuals. A result that could also be confirmed with a facial EMG study. This finding is in line with notions from attachment theory as well as that secure individuals display more happiness in their facial expressions and thus more emotional resources. The latter hypothesis was also confirmed in another study. Here, too, an interaction between attachment and psychopathology was found. Secure healthy individuals not only showed more happiness in their facial expressions than insecure individuals but also more than secure individuals with anxiety disorder. However, another study could not confirm the correlation between facial displays of happiness and secure attachment. Here, no difference was found between secure and insecure-dismissing individuals. However, consistent with attachment theory, insecure-preoccupied individuals did display more facial expressions of unpleasant emotions. The theoretically consistent attachment hypothesis that securely attached individuals both respond more resourcefully to attachment-related stressors and already experience attachment as an emotional resource was also shown by a study examining the hand-blink reflex (HBR) as a subcortical defense response of the peripersonal space (PPS). In the fourth FACS study, due to the small sample size, only the difference between a disorganized–unresolved and an organized–resolved attachment representation was examined. While unresolved borderline patients displayed more disgust in their facial expressions, unresolved healthy individuals facially expressed more contempt. Thus, as a superordinate cluster linking these two emotions, activated avoidance motivation is found here for the unresolved attachment group.

In summary, studies on attachment-related differences in facial behavior, thus, imply that secure-attached individuals display more emotional resources in the form of happiness in response to attachment-related stressors, and insecure-dismissing individuals are less moved facially and overall nonverbally. Further studies, especially with larger sample sizes and more comprehensive analysis of facial behavior, are needed to draw clear conclusions about the other correlates. Optimally, future studies will include other nonverbal channels beyond facial expressions, such as gestures, body posture, and head posture. This is because studies indicate that when more than facial expressions are included, even more emotions can be detected in nonverbal behavior beyond the originally formulated basic emotions [146,147]. This is especially relevant for social emotions such as love, pride, shame, and embarrassment [86,87,148,149,150]. Additionally, these could play a special role in attachment-related differences in affective expression because attachment is, per se, a social phenomenon.

The two studies that examined attachment-related differences in vocal behavior in response to attachment-related stressors confirmed, first, as well, that secure individuals have more attachment-related resources. This was shown by the fact that the more parental rejection subjects had experienced in childhood, the larger the difference between secure and insecure-dismissing subjects in the degree of coherence between the valence of verbal responses in the AAI and the subjects’ vocal affective cues. This showed the ability of secure individuals to emotionally reprocess and coherently reorganize negative childhood experiences. Second, both vocal behavior studies found that insecure-dismissing individuals showed generally higher levels of incoherence between verbally expressed emotional experience and vocally unconsciously expressed stressful emotion compared to secure individuals. For example, insecure-dismissing individuals tended to downplay negative childhood experiences but revealed vocal signals that were indicative of stress. Both results are consistent with attachment theory considerations.

One study examined attachment-related differences in marital interaction behavior and showed, first, that insecure compared to secure attachment is associated with more destructive conflict behavior in the partnership. This again indicates that securely attached individuals have better access to their emotional resources in attachment-related situations. Second, insecure-dismissing husbands showed more stonewalling and contempt in a conflict with their wives. Both behavior patterns are displays of an attachment-avoidance motivation and thus can be considered consistent with attachment theory for insecure-dismissing individuals. This result is one possible explanation for why insecure attachment predicts both a history of divorce and single relationship status [53,151].

#### 4.1.5. Summary of Results for All Four Subgroups

In the following, we summarize attachment-related differences in emotion regulation, not from the perspective of the four approaches to objective measurement of emotion regulation, but with respect to the four distinct attachment groups in adults: secure, insecure-dismissing, insecure-preoccupied, and unresolved.

Current research clearly indicates that a secure attachment representation is associated with adaptive emotion regulation and better access to one’s emotional resources. This is also shown in the form that the attachment system, per se, is already experienced as a resource that promotes healthy stress coping. This finding is consistent with attachment theory and confirms the hypothesis formulated at the beginning that secure attachment is associated with balanced emotion regulation. Experiencing attachment as a resource also explains why securely attached individuals tend to feel emotionally connected to others even when they are currently alone, which in turn reduces feelings of loneliness.

In contrast, insecure attachment in general, but disorganized–unresolved attachment in particular, correlates consistently with attachment theory notions with increased attachment avoidance motivation, according to the current body of research across all four measurement approaches to emotion regulation. It is associated with maladaptive emotion regulation in response to attachment-related stressors compared to secure attachment. This also confirms the hypothesis formulated at the beginning of this systematic review that emotion regulation is impaired in insecure attachment and dysfunctional in disorganized–resolved attachment.

The insecure-dismissing attachment representation seems to be associated with heightened arousal, i.e., heightened activation of the sympathetic nervous system, in response to attachment-related stressors. At the same time, the studies for this attachment classification are equally indicative of increased activation of avoidance motivation and emotional withdrawal. This is shown both in less moved and less expressive nonverbal behavior and in increased incoherence between subjective feelings and objectively measurable stressful emotions. For example, consistent with attachment theory, negative childhood experiences are downplayed in their emotional significance, while at the same time, clear stress signals are observed on several measurement pathways. This finding confirms the hypothesis formulated at the beginning that insecure-dismissing individuals tend to try to downregulate their emotional response to attachment-related stressors as much as possible. However, as the studies included in this systematic review show, they do not fully succeed in doing so. Thus, facial expressions seem to be more motionless and controlled, while at the same time, emotional stress is still recognizable on a physiological and biochemical level as well as in vocal behavior. Here, it would be exciting to investigate the occurrence of microexpressions in future studies regarding the facial behavior of insecure-dismissing persons. Microexpressions are emotionally involuntarily elicited facial expressions that are found only for fractions of a second (≤500 ms) in partial areas of the face. According to Haggard and Isaacs [152], these show mainly emotions that the person is not aware of or wants to suppress (see also [91,153,154]).

To make clear statements about the typical patterns of emotion regulation for insecure-preoccupied individuals, a sufficient number of studies is lacking. Nevertheless, there is a preliminary indication, consistent with attachment theory, that this attachment classification is associated with increased stressful emotions and increased display of unpleasant emotions in the context of attachment-related stress. This confirms the hypothesis formulated in the introduction section that insecure-preoccupied individuals tend to upregulate their emotional response to attachment-related stress in order to communicate it clearly to the outside world.

### 4.2. Theoretical Implications

In accordance with Mikulincer and Shaver [41], we may conclude from our results that attachment security is connected with constructive regulations of emotions by demonstrating the ability to be aware and perceive one’s own emotional distress combined with the flexibility and inner freedom to think about attachment-related events without fear of being overwhelmed or losing control. When confronted with attachment-related distress secure individuals show adaptive emotion regulation on different objective levels (autonomic nervous system, brain activity, biochemistry, or nonverbal behavior). On a representational level, they are characterized by the capacity to refer to an internalized secure base, a haven of safety, or constructive capacity to act [32] and a coherent sense of self [30] with the ability to forgive and constructively value the impact of attachment experiences on their personal development.

Dismissing attachment is connected with the inhibition of emotional experience in order to block or avoid potentially painful associations with rejection and abandonment from significant others. In our review, these deactivating strategies are demonstrated on different objective levels and cause individuals to avoid noticing their own emotional reactions. Bowlby [19] described avoidant inhibition and denial of emotional experiences in terms of defensive exclusion (deactivation). On a representational level, these individuals are characterized by emphasizing personal strength and functional relationships with a limited ability to refer to an internalized secure base. The impact and meaning of attachment experiences on personal development tended to be diminished and devaluated.

In contrast, preoccupied attachment is connected with an intensification of undesirable emotions since these individuals have internalized that hyperactivation of attachment secures the mental closeness to the attachment figure. On a representational level, these individuals are characterized by ambivalence, insecurity, vagueness, and a chaotic mental architecture pervaded by unpleasant emotions. This idea is also compatible with the preliminary results of our systematic review.

Although deactivating and hyperactivating strategies lead to contrasting patterns of emotions, both result in maladaptive emotional experiences associated with impaired capacities for flexible mental exploration.

Specifically unresolved attachment is associated with a dysfunctional management of attachment-related threats. When confronted with attachment-related issues, they demonstrated the highest signs of emotional distress on different objective levels. On a representational level, these individuals are characterized by a breakdown of strategies to contain and reflect attachment-related threats such as loss, maltreatment, and emotional neglect and are not able to find constructive resolutions [32]. In contrast to deactivating and hyperactivating strategies in organized insecure individuals, unresolved ones fail to tolerate and integrate overwhelming emotions.

### 4.3. Practical Implications for Psychotherapy and Coaching

Analysis of the current body of research on the correlation between individual attachment representation and patterns of emotion regulation shows that there is a clear link between the two. Research demonstrates impaired dysfunctional emotion regulation for both insecure and disorganized–unresolved attachment representations. Considering the increasing trend in the prevalence of mental disorders, this is an important notion. A large body of studies has already shown that attachment prevention programs can be effective in strengthening parent–infant attachment in terms of secure attachment (e.g., [155,156,157]). Similarly, several studies indicate that psychotherapy positively affects and changes attachment representation in adults (for a review, see, e.g., [158]). This was even evidenced in patients with borderline personality disorder [159] or severe major depression [34] during psychodynamic psychotherapy when focusing on internalized representations of significant others in the therapeutic relationship.

Further studies are warranted to examine the mechanisms of change during prevention and intervention by analyzing the individuals’ emotional regulation processes using a broad spectrum of measures such as nonverbal behavior, brain activity, biochemistry, and autonomic nervous system activity (see four approaches in Section 1.2). One of the possible starting points here would be the central link between oxytocin and a secure attachment representation. A correlation that also Buchheim et al. [160] could demonstrate. In their study, male study participants, all previously classified by the AAP as insecurely attached, were administered intranasal oxytocin. The oxytocin resulted—at least for that moment—in the subjects feeling their attachment representation was more secure or showing more willingness to be attached. Researchers already knew by then that oxytocin plays a central role in the experience and behavior of our interpersonal relationships, especially prosocial behavior [161]. However, this was the first study to demonstrate that it also directly influences the perceived quality of attachment representation. This notion could have promising applications in psychotherapy or even in individual coaching aimed at healthy individuals. For example, the research found that gratitude is closely linked to the oxytocin system [162]. In addition, studies demonstrated that when gratitude is emotionally activated through a brief daily exercise, it decreases subjective feelings of loneliness [163]. In another experiment, researchers asked their subjects to perform the following: “Please think back to last week and write down five things you are grateful for in your life”. The subjects then resulted in performing this exercise once a week for a period of ten weeks. As a result, the study participants felt more satisfied with their lives in general, more optimistic regarding the next week, and—this is crucial for us here—also more emotionally connected to other people [164]. Thus, a gratitude intervention can reduce feelings of loneliness and increase feelings of connectedness to others. Here, it would be interesting to conduct studies that examine the extent to which emotionally cultivating gratitude in one’s daily life can strengthen the quality of attachment representation. After all, this would be an intervention that is easy to integrate into everyday life and that can be used in coaching as well as in psychotherapy.

Building on the notions of this review, recommendations can be derived for the approach in psychotherapy and coaching regarding the different types of attachment. Since insecure-dismissing individuals tend to use emotional deactivation strategies, i.e., to suppress unpleasant emotions, treatment techniques that help the client to encounter his or her feelings of stress in a mindful and controlled manner are particularly recommended. Studies could show, for example, that a guided conscious perception and judgment-free acceptance of one’s own feelings through a body scan promotes interoception and emotional sensation accuracy [165]. At the same time, a body scan affects the decrease in stress hormone levels cortisol [166]. In contrast, for the typically emotionally hyperactivating insecure-preoccupied individuals, i.e., those who tend to upregulate unpleasant emotions, emotion regulation techniques are recommended to keep exposure within the “window of tolerance” during an intervention. According to Siegel [167], in the “window of tolerance”, a person can experience emotions of various types and intensities without disrupting healthy functioning. This is where learning and development take place and, thus, where change occurs during psychotherapy or coaching. The emotions should therefore be felt in an intervention, not too intense but balanced. An effective starting point for an easy-to-use emotion regulation technique is slow-paced breathing (e.g., inhale for 5 s and exhale for 5 s). Thus, studies report that even a short sequence (e.g., 5 min) of slowed-paced breathing improves performance on executive function tasks (e.g., Stroop task) [168], increases HRV, enhances a sense of relaxation [169], and reduces performance anxiety [170]. If the therapist or the coach, respectively, notices that the client is overwhelmed by their emotions during an intervention, they can quickly and easily help the client to regulate the emotional stress by using guided slow-paced breathing. In disorganized–unresolved individuals, due to the presence of dysfunctional emotion regulation, special attention should be paid to the “window of tolerance”. In this case, it is advisable to create additional settings that increase the emotional security of the client. This can be achieved, for example, at the beginning of a session by the guided imaginative activation of a secure place (e.g., [171]) or by establishing contextual security signals. For the latter, ask the client, for example, “What things here in this room make you feel secure?” or the client may bring an object to the session that makes her or him feel secure. Research showed that establishing such safety signals in the current context helps to immediately reduce feelings of anxiety [172].

### 4.4. Risk of Bias in Included Studies

Limitations of the specific included studies were described in the Results section. Here, we summarize the limitations from an overall perspective. The first limitation that can be found is that most of the subjects (67%) are women. This limits the generalizability of the results for men. Similarly, a potential risk of bias is that 70% of the studies were conducted in Germany or the United States. The other studies were also conducted in Western cultures, which raises the question of the extent to which the results can be transferred to other cultures. Similarly, in many of the studies, the sample size was too small to examine differences in emotion regulation across all four attachment groups. For example, no clear conclusions can be drawn for insecure-preoccupied individuals because this attachment subgroup was usually represented in insufficient numbers in the studies. Likewise, insecure attachment classifications were combined into one category in many studies for reasons of too small a sample size. Sometimes, even “only” organized attachment was examined in contrast to disorganized attachment in its differences in emotion regulation. Overall, the attachment-related stressors used were very heterogeneous. Here, there was no superordinate common categorization of the types of attachment-related stress. This makes the comparison between different studies and, thus, a summary analysis more difficult. For example, the interpretation of the HRV response as adaptive or maladaptive may differ depending on whether coping with the stressor involves executive function or not [58]. One suggestion here would be to divide attachment-related stressors into three categories, each with two possible manifestations. First, a stressor can be divided into a stimulus with a concrete personal relation (e.g., the questions of the AAI or the cry of one’s own infant) versus an ambiguous stimulus which the subject may potentially relate to her-/himself (e.g., AAP pictures or the cry of an unfamiliar infant). The second category is in sensu versus in vivo. An in sensu stimulus takes place only in the client’s imagination (e.g., the imagination of a conflict situation). An in vivo confrontation, on the other hand, consists of the perception of the stressor living in the situation with the external senses (e.g., a live conflict resolution task with a romantic partner). The third category consists of the distinctness of the valence of the stimulus. Thus, stimuli can be divided into unambiguously stress-inducing or unambiguously negative in valence (e.g., the cry of an infant) or potentially stress-inducing (e.g., a picture of one’s mother). This 3 × 2 categorization results in eight types of attachment-related stress that could be successively investigated via studies. For example, the AAP could be seen as an ambiguous stimulus, which is administered in vivo and is potentially stress-inducing. In contrast, the imagination of a social conflict has a concrete personal relation, is in sensu, and is unambiguously negative in its emotional valence.

As described in Section 2.3., we controlled the risk of biases in the included studies by applying a modified checklist of four items derived from Busse and Guyatt [104]. However, in general, we should state that we cannot rule out that our included studies may overestimate effects because we have not included unpublished results, and therefore, we could not exclude a possible publication bias. The reason for this selection was our focus on peer-reviewed articles. Therefore, we were not able to compare the results of published and unpublished papers, which can be considered a limiting factor.

### 4.5. Future Research in the Lens of Results

Some ideas for future research arising from the analysis of the studies included in this systematic review have already been elaborated in the section on the interpretation of results. Here, we now consider four superordinate ideas to fill existing research gaps.

First, it is desirable that future studies use a larger sample size. Only then will it be possible to examine and statistically analyze differences in emotion regulation across all four attachment classifications. One bottleneck here is the time required to measure individual attachment representation. Here, the AAP is very promising, as it allows a valid and reliable measurement of attachment representation in a mean of 30 min. Likewise, the nonverbal observation channel is promising here, as facial expressions and body language are immediately recognizable and thus easy to use. Both approaches are also well usable for psychotherapists and, likewise, coaches in a conversation with the client. For the area of the nonverbal, however, it must be emphasized that further research is needed here to make clear statements about attachment-related differences and individual defensive processes. On the other hand, there is another bottleneck in the time or financial effort regarding the objective measurement of the emotional response. To be able to analyze larger numbers of subjects in a time-efficient way, research on the automated measurement of nonverbal behavior can be intensified, for example.

Second, more men (respectively fathers) should be included in future studies, as they are currently underrepresented as subjects in attachment research [173]. This is an important point since fathers are playing an increasingly prominent role in parenting today. Likewise, studies indicate that fathers, like mothers, have an important function in the emotional development of their infants (e.g., [174]).

Third, it would be exciting to combine different ways of measuring emotional responses even more frequently. Thus, it would be possible to investigate the degree of coherence between different emotional response systems. Furthermore, a more integrated picture of attachment-related differences in emotion regulation can be obtained this way. There are already studies that showed that individual indicators are interrelated. For example, SCL seems to correlate with frontal right asymmetry and amygdala activation [175,176,177]. If reliable correlations can be discovered here, cost-effective measurement methods could be relied upon, and more expensive measurement pathways that may indicate the same type of emotional response could be avoided. In turn, this would make it possible to analyze larger sample sizes.

Fourth, the studies analyzed in this review showed that it may be important in examining attachment-related differences in emotion regulation to consider the interaction of attachment with potentially present psychopathology. Therefore, it is recommended for future studies to control for psychopathological symptoms as a standard part of the study design. In this way, it can be analyzed more precisely what influence the individual attachment representation has on the ability to regulate emotions.

## 5. Conclusions

Our systematic review focused on differences in emotion regulation responses in individual attachment representations. These were measured via the Adult Attachment Interview or Adult Attachment Projective Picture System, which are established narrative instruments to assess adult attachment on an unconscious level. Emotion regulation in these studies was measured via one of the four following objective approaches: autonomic nervous system, brain activity, biochemistry, or nonverbal behavior.

Across all measurement approaches, the results reveal a significant correlation between individual attachment representation and emotion regulation patterns. In sum, secure attachment correlates consistently with more balanced and adaptive emotion regulation, whereas emotion regulation is impaired in insecure and dysfunctional in disorganized–unresolved attachment. Specifically, unresolved individuals display counterintuitive responses and fail to use attachment as a resource. Insecure-dismissing attachment is associated with an emotionally deactivating strategy, while on a physiological, biochemical, and nonverbal level, the emotional stress is still present. There is still a lack of studies examining preoccupied individuals. These results imply specific recommendations for attachment-informed interventions in psychotherapy and coaching.

What did we learn from our results when working with individuals with different attachment representations and emotion regulation strategies? Since individuals with secure attachment representation show a balanced and open way to cope with various feelings on different levels, we may conclude that these individuals will be collaborative and compliant in treatment and coaching. Insecure-dismissing individuals show emotionally deactivating strategies but remain emotionally stressed on a physiological level. This goes along with their tendency to view themselves as independent, strong, and self-sufficient. Working with these individuals, we may anticipate that they become more distressed and reject when confronted with emotional issues. In contrast, preoccupied individuals are interpersonally overengaged and hyperactivate their attachment-related emotions such as anger and rage. Preoccupied clients tend to present themselves as needy and might be more clingy in the therapeutic relationship. Unresolved individuals are not able to contain and integrate feelings of attachment-related threat and danger and show emotional dysregulation when confronted with these issues. Therefore, the target of treating these clients should focus on the reorganization of emotional distress by working on the disrupted attachment-related representations of self and significant others.

This systematic review shows that the ability to regulate emotions, which is essential for the outcome of psychotherapy, is substantially correlated with the client’s attachment pattern. Therefore, our recommendation is to always consider attachment in the context of psychotherapy and coaching, possibly even by measuring the individual’s attachment representation at the beginning of treatment. This makes it possible to adapt the concrete therapeutic procedure to the client’s attachment pattern in the sense of attachment-informed psychotherapy or attachment-informed coaching, respectively.

## Figures and Tables

**Table 1:** Overview of response systems, measures, and emotional states to which they are sensitive.

Response System	Measure	Sensitivity
Peripheral physiology	ANS measures	Arousal (and negative valence)
Central physiology	EEG	Approach and avoidance
	fMRI, PET	Approach and avoidance
Biochemical level	Saliva and blood samples	Approach and avoidance
Behavior (nonverbal)	Facial behavior coding	Specific emotions and valence
	Facial muscle activity (EMG)	Valence
	Vocal characteristics	Arousal

**Table 2:** Keywords of the search strategy according to the PICO approach.

PICO Component	Search Syntax (Keywords)
Population	(adult* OR adolescent*)
“Intervention” (emotion regulation of attachment-related stress)	(emotion regulation OR affect regulation OR distress OR dysregulation)
Comparator (differences in attachment representation)	(attachment representation OR attachment pattern* OR attachment classification* OR aai OR aap OR adult attachment interview OR adult attachment projective)
“Outcome” (measurement of emotion regulation)	(neuroimaging OR neuro imaging OR neuroscience OR brain activity OR fmri OR eeg OR electrodermal activity OR skin conductance OR cortisol OR oxytocin OR neuroendocrine OR blood pressure OR heart rate variability OR hrv OR heart frequency OR facial expression* OR gesture* OR facs OR facial action coding system)

* These are truncations and part the search syntax.

**Table 3:** PICO components.

PICO Component	Inclusion Criteria
Population	All adults and adolescents (≥12 years), non-clinical and clinical population
“Intervention”/exposure	Emotion regulation of attachment-related stress
Comparator	Differences in attachment representations, measured by Adult Attachment Interview (AAI) or Adult Attachment Projective Picture System (AAP), concretely secure, insecure-dismissing, insecure-preoccupied, unresolved
“Outcome”	Four objective measures of emotion regulation (measurement of central physiology, peripheral physiology, biochemical level, or nonverbal behavior)
